# Identification of Th1/Th2 regulatory switch to promote healing response during leishmaniasis: a computational approach

**DOI:** 10.1186/s13637-015-0032-7

**Published:** 2015-12-01

**Authors:** Piyali Ganguli, Saikat Chowdhury, Shomeek Chowdhury, Ram Rup Sarkar

**Affiliations:** 1 Chemical Engineering and Process Development Division, CSIR-National Chemical Laboratory, Pune, Maharashtra 411008 India; 2 Academy of Scientific & Innovative Research (AcSIR), CSIR-National Chemical Laboratory, Pune, India

**Keywords:** T cell and APC signaling pathways, *Leishmania*, Th1/Th2 response, NO synthesis, Logical model, Immunotherapy, Combinatorial drug targets

## Abstract

*Leishmania* devices its survival strategy by suppressing the host’s immune functions. The antigen molecules produced by *Leishmania* interferes with the host’s cell signaling cascades and consequently changes the protein expression pattern of the antigen-presenting cell (APC). This creates an environment suitable for the switching of the T-cell responses from a healing Th1 response to a non-healing Th2 response that is favorable for the continued survival of the parasite inside the host APC. Using a reconstructed signaling network of the intracellular and intercellular reactions between a *Leishmania* infected APC and T-cell, we propose a computational model to predict the inhibitory effect of the *Leishmania* infected APC on the T-cell and to identify the regulators of this Th1-/Th2-switching behavior as observed during *Leishmania* infection. In this work, we hypothesize that a complete removal of the parasite could only be achieved with a simultaneous up-regulation of the healing Th1 response and stimulation of nitric oxide (NO) production from the APCs, and downregulation of the non-healing Th2 response and thereby propose several unique combinations of protein molecules that could elicit this anti-*Leishmania* immune response. Our results indicate that TLR3 may play a positive role in eliciting NO synthesis, while TLR2 may be responsible for inhibiting an anti-*Leishmania* immune response. Also, TLR3 overexpression (in the APC), when combined with SHP2 inhibition (in the T cell), produces an anti-*Leishmania* response that is better than the conventional IFN-gamma or IL12 treatment. A similar anti-*Leishmania* response is also obtained in another combination where TLR3 (in APC) is overexpressed, and SHC and MKP (of T cell) are inhibited and activated, respectively. Through our study, we also observe that *Leishmania* infection may induce an upregulation of IFN-beta production from the APC that may lead to an upregulation of the RAP1 and SOCS3 proteins inside the T cell, the potential inhibitors of MAPK and JAK-STAT signaling pathways, respectively, via the TYK2-mediated pathway. This study not only enhances our knowledge in understanding the Th1/Th2 regulatory switch to promote healing response during leishmaniasis but also helps to identify novel combinations of proteins as potential immunomodulators.

**Electronic supplementary material:**

The online version of this article (doi:10.1186/s13637-015-0032-7) contains supplementary material, which is available to authorized users.

## Introduction

Cell-mediated immunity (CMI), responsible for confronting the infections caused due to invasion of intra-cellular pathogens, primarily involves the interactions of the phagocytic antigen-presenting cells (APCs) and the T-lymphocytes. This leads to the activation of a series of intra-cellular and inter-cellular biochemical signaling processes, which culminates into synthesis of certain diffusible effector molecules that includes proteins (mostly the cytokines) and microbicidal molecules (e.g., nitric oxide) helping in the clearance of the disease [1]. However, the activities of this defense mechanism are severely compromised during leishmaniasis, a neglected tropical disease, caused due to infection by the protozoan parasites of the genus *Leishmania*. This is transmitted to the human through the infected bites of the phlebotomine sand flies during their blood meal [1]. The promastigote form of the parasite, once injected into the human host, is engulfed by the APC (macrophages and dendritic cells) to form a phagolysosome, where it differentiates into its amastigote form and takes control of its entire cellular machineries in a way that reduces the immuno-competency of the immune cells thereby hindering the body’s natural parasite clearance process [2].

The surface molecules produced by *Leishmania*, such as, lipophosphoglycan (LPG), glycoprotein 63 (GP63), and the elongation factor EF1-alpha directly or indirectly activate a series of phosphatases inside the human APCs (e.g., SHP1, PTP1B, and TCPTP), that leads to dephosphorylation and de-activation of important signaling molecules inside the host cell [3]. Inside the APC, the LPG molecules act as antigens and are presented to the surrounding T-lymphocytes to elicit either of the two types of immune responses, *viz.* healing and non-healing responses, depending on the parasite load and the host immunity [4]. The healing response is obtained in case of low parasitic load, in which a pronounced Type-I helper T-cell (or Th1) response occurs due to up-regulation of the Th1 cytokines, such as the interferon-gamma from the stimulated T cells, and thus naturally clears the pathogen from the system [1, 5]. On the other hand, higher pathogen load gives rise to a non-healing response in which an upregulation of the Th2 cytokines (e.g., IL10) is observed, that favors the persistence of the *Leishmania*. Simultaneously, during this non-healing response, the production of the protective Th1 cytokines, such as IL12, and the microbicidal molecules, such as nitric oxide is also downregulated, thus creating an immune-suppressed condition suitable for the further progression of the disease [6].

It is experimentally shown that all types of leishmaniasis viz. cutaneous, muco-cutaneous, and visceral leishmaniasis elicit these types of immune responses in human body [7]. Hence, the general therapeutic strategy adopted for the treatment of Leishmaniasis is primarily aimed to expedite the process of parasite clearance for faster healing by stimulating the Th1 or healing response. In case of cutaneous leishmaniasis therapeutics, chemotherapeutic drugs, such as pentavalent antimonials, liposomal amphotericin B has been shown to be useful to reduce the dermal lesions and the chances of further destructive mucosal inflammations and visceral infections [8, 9]. However, the successive clinical studies have shown that these chemotherapeutic drugs are also associated with adverse side effects, such as nausea, intense headache, diarrhea, musculoskeletal and abdominal pain etc. [9–13]. In several cases, relapse of the disease and developing resistant strains are also reported after the use of these drugs, which necessitates the development of better treatment protocols with higher clinical efficacy [14]. Although immunotherapeutic strategies involving the administration of exogenous interferon-gamma is found to be effective in suppressing leishmaniasis [15, 16], the high production of IL10 during early stage of infection often suppresses its activity, thereby hindering NO production and disease clearance [17]. Based on these experimental outcomes, a number of mathematical models have also been proposed simultaneously to untangle the complexities that appear as hurdles to device a successful treatment strategy in leishmaniasis [18, 19]. In one of such studies, “granulomas” formation during *Leishmania donovani* infection has been modeled using Petri net analysis by considering the inter-cellular interactions of macrophage, lymphocyte, NK cells etc. The outcomes of these cell population based models have emphasized cytokine therapy by the exogenous injection of interferon-gamma and the suppression of IL10 to eradicate the *Leishmania* pathogens in macrophage cell [20]. However, interferon-gamma molecule is a pro-inflammatory molecule and also has short half-life time, which in turn requires its repeated administration into the body at a regular interval of time that may have harmful consequences [21, 22]. Hence, to circumvent these problems, implementation of better therapeutic strategies, by identifying novel drugs, drug target molecules and immunostimulators are required and demands higher attention from the vast majority of clinical and experimental pharmacologists.

However, in order to develop an effective immunotherapeutic strategy, it is important to have a comprehensive understanding of the Th1/Th2 dichotomy in leishmaniasis so as to identify the regulators through which the Th1/Th2 switching behavior can be effectively controlled. This mechanism still remains very less explored. The identification of such important molecular switch and their corresponding reaction routes through which the immunostimulation could be enhanced is highly required in this field of study. As the exact intra-cellular reaction cascades governing the T cell response after encountering with *Leishmania* infected APCs is not clearly understood yet, the mechanisms through which this response dynamics and the nitric oxide (NO) production work in the immune cells is still unknown. Besides, the mechanism through which the *Leishmania* antigens override the APCs intra-cellular network by varying the expressions of the immunostimulatory proteins, and force to redirect the immune responses towards the non-healing or Th2 response is not comprehensively studied yet. The study of these regulatory mechanisms by analyzing such a large system using conventional experimental techniques is time consuming and also difficult to perform, and therefore in silico mathematical models of inter and intra-cellular reaction cascades in APC and T cell in presence of *Leishmania* antigens would probably be the best strategy to counteract these problems. This may also help to address some of the unexplored questions of *Leishmania* immunotherapy, such as the limitations of the interferon-gamma treatment, the reason for which interferon-beta treatment is only effective at low doses, and the means by which the toll-like receptor (TLR) molecules expressed by the APCs can regulate the immune responses of the T cell to shift the dynamics towards a higher healing Th1 response [17, 23–25].

In this study, we have tried to address the above mentioned problems in *Leishmania major* infection scenario by using mathematical model and *in silico* analysis. We have hypothesized that in order to achieve better therapeutic results without adverse side effects, the stimulation of type-I T-helper cells and a simultaneous upregulation of NO production by using immunostimulator would be the best therapeutic strategy to clear the *Leishmania* pathogens from the body. In order to develop a suitable in silico model that may enhance our understanding of *Leishmania* immunobiology, we have manually reconstructed a comprehensive cell signaling pathway map of a *Leishmania* infected APC and a normal CD4^+^ T cell (helper T cell), considering the important physical interactions and the cross-talks by the secreted diffusible molecules between the two cells. The *Leishmania* infection has been introduced in the model by establishing the interaction of the *Leishmania* antigens, known from the literature and databases, with the appropriate host protein molecules in the APC. However, the dynamic analysis of such a large network is difficult to perform due to the unavailability of kinetic parameters and concentration values. Hence, to assess the gene or protein expression patterns of large scale signal transduction networks under different pathological conditions, the concept of discrete dynamic Boolean or logical modeling approach has been utilized successfully [26–28]. Large scale, intracellular T cell signaling network is also analyzed by using this modeling technique and eventually various structural and functional properties of this network under normal and disease conditions are studied successfully [29, 30]. A logic-based modeling technique is also applied to analyze the temporal expression patterns of the genes/proteins of T cell, which are strongly influenced by the intra-cellular T cell signal transduction cascade in presence or absence of infection [31].

Here, the entire reaction mechanisms are translated into logical equations with the objective to simulate and understand the effect of the presence and absence of the *Leishmania* antigens on the signaling events of the host’s APCs and T cells. Followed by the Boolean attractor analysis and the successful validation of the simulation outcomes with the time-course microarray expression data as well as the phenotypic responses obtained from published experimental observations, the model is then used to compare the protein expression pattern for normal and *Leishmania*-infected scenarios. With an aim to understand the mode of regulations that occur due to the infection at the molecular level inside the T cell, the comparison of the two scenarios is then used to extract the important T cell proteins, which are highly influenced under the pathogen burden. The result of this analysis is further used to predict the unknown changes occurring at the pathway level in the T cell during infection. Moreover, the knowledge of these deregulated pathways is thereafter used to predict the targets for the *in silico* perturbation analysis. Perturbations of the logical states of proteins in the network are performed to study the effect of the known immunostimulants (*viz.* IL12 and interferon-gamma) as well as to propose some new combinations of molecules that act as a molecular switch to regulate the Th1/Th2 and NO response dynamics. Subsequently, these identified novel combinations of proteins were tested for stability and robustness by examining the attractors of the system under these perturbations. Thereafter, it was ascertained that the proposed combinations of protein targets can be used as the potential immunomodulators, targeting of which may bypass the inhibitory activities of the pathogens and enhance the anti-*Leishmania* immune responses as well as the microbicidal activities of the body’s immune cells.

## Materials and methods

### Construction of gene correlation network

Gene correlation networks of the significantly expressed genes, observed in two independent microarray experiments for APC (E-GEOD: 42088) and T cell (E-GEOD: 48978), were constructed in this work by calculating the Pearson correlation coefficient of each pair of genes from the temporal gene expression data followed by the calculation of *P* values. The *P* values of all pair of genes from the two microarray data sets were stored in symmetric square matrices from which the corresponding adjacency matrices were generated. In the adjacency matrices, the elements are either 1 (*P* value < 0.01) or 0 (otherwise). These adjacency matrices are then used for the construction of co-expression or correlation networks of the two microarray gene expression datasets. The networks are then analyzed for the identification of probable clusters (or functional modules) in Cyctoscape (version 2.8) GPU based App AllegroMCODE (version 2.1) [32]. The genes from each functional module identified in this analysis are further used for the pathway enrichment analysis in bioCompendium (http://biocompendium.embl.de/) and GeneCodis [33] web servers [Additional file 1: Text S1/Table S1 and Text S2/Table S2]. The pictorial representations of each cluster are provided in Additional file 2: Figure S1 and Additional file 3: Figure S2.

### Pathway reconstruction/integration

In order to capture the functional regulations that operate between these significantly enriched pathways within the two cells, i.e., APC and T cell, reconstruction of a comprehensive map of signaling processes depicting the effect of *Leishmania* infection on immune response was necessary. Hence, a detailed T cell and APC interaction pathway diagram was created after a thorough study of existing literatures and databases. Protein-protein interaction (PPI) and the biochemical signal transduction data were collated from various cell signaling and PPI databases, such as KEGG, Protein Lounge, Pathway Central, Biocarta, NetPath, BIOGRID, etc. and various published research articles [34–37]. The *Leishmania* proteins were then introduced in the network and the interactions of these proteins were established with the existing APC molecules depending on the biological evidences [38–40]. The *Leishmania* antigenic molecules used in the model, viz. LPG_L, GP63_L, LFAA_L, and EF1_ALPHA_L, are known to be present in almost all the *Leishmania* species so as to create a generalized *Leishmania* infection model (LFAA_L is a hypothetical molecule which we considered in our model to show the activation of ASMASE for the production of CERAMIDE [41]; it is abbreviated for *Leishmania* factor activating ASMASE). With certain modifications (required to build the juxtacrine and paracrine interactions between the cells), the T cell pathway reported in our previous work was used to understand the T cell-APC cross-talks and to monitor the immunological response generated during *Leishmania* infection [31]. The pathway figure was drawn using Cell Designer software (version 4.3) [42]. The signaling molecules (nodes) and interactions were color coded in accordance with cellular locations and their chemical nature, respectively. Also, in order to differentiate the redundant *Leishmania* and T cell molecules from the APC molecules, the names of the protein/non-protein molecules were denoted with suffix “L” and “T” for *Leishmania* and T cell, respectively (Additional file 4: Figure S3 and Additional file 1: Text S3).

### Model formulation

The interactions of the entire network, including all important regulations between T cell and APC, were translated into logical equations (signifying reactions or hyper acrs) using the *AND*, *OR,* and *NOT* logical gates, in a biologically meaningful way (Additional file 1: Text S5). In order to capture the regulations at the post-transcriptional level, the alternatively spliced isoforms of the T cell and APC output molecules with known functions have been also included in our model (Additional file 1: Text S4/Table S3). Here, the selection of isoforms is based on the presence of certain cis-regulatory elements and trans-acting factors that have been collectively referred to as “FACTORi” where {*i* = 1,2,.....23}. These 23 FACTORi represent specific spliceosomes responsible for the splice site recognition in each case.

The model was simulated synchronously (i.e., all equations updated simultaneously) and asynchronously (i.e., random execution of the equations) using BooleanNet-1.2.4 software until the steady state is reached [43]. In this model, we also defined three functions, viz. “TH_1_response*”, “TH_2_response*”, and “NO_response*”, which reflect the type of T cell responses elicited and production of NO from the APC in response to an infection (Eqs. 1, 2, and 3; * denotes the (t+1)^th^ logical state of the responses). The molecules used for defining these functions are principally the molecules involved in eliciting these responses, as reported in literatures [44].

1 $$ \mathbf{T}\mathbf{H}\_\mathbf{1}\_\mathbf{response}*=\mathbf{I}\mathbf{L}\mathbf{2}\_\mathbf{T}\; AND\;\mathbf{G}\mathbf{M}\_\mathbf{C}\mathbf{S}\mathbf{F}\_\mathbf{T}\; AND\;\mathbf{T}\mathbf{N}\mathbf{F}\_\mathbf{ALPHA}\_\mathbf{T}\; AND\;\mathbf{I}\mathbf{F}\mathbf{N}\_\mathbf{GAMMA}\_\mathbf{T} $$

2 $$ \mathbf{T}\mathbf{H}\_\mathbf{2}\_\mathbf{response}*\kern0.37em =\mathbf{I}\mathbf{L}\mathbf{4}\_\mathbf{T}\; AND\;\mathbf{I}\mathbf{L}\mathbf{5}\_\mathbf{T}\; AND\;\mathbf{I}\mathbf{L}\mathbf{6}\_\mathbf{T}\; AND\;\mathbf{I}\mathbf{L}\mathbf{10}\_\mathbf{T} $$

3 $$ \mathbf{NO}\_\mathbf{response}*=\mathbf{NO} $$

### Boolean attractor, experimental data and validation

The Boolean attractors of the model were determined by generating all possible combinations (ON or OFF) of the 51 input molecules of the system. The simulation was repeated for 20 samples, where 7 proteins have been selected from a uniform random distribution of 51 input molecules, thereby generating 2^7^ × 20 (=2560) combinations of input molecules. However, due to lack of human cell-specific *Leishmania major*-infected RNA seq data of APC, the logical states (activation or inactivation) of the FACTORi determining that alternative splicing of the output molecules could not be explicitly determined in *Leishmania*-infected scenario. Hence, in our model, these FACTORi were assumed to be ON in all our simulations, signifying that all the alternative isoforms have equal probability of getting expressed. The analysis was performed separately for the uninfected and the infected scenarios, which were created by initializing the *Leishmania* antigen molecules OFF and ON, respectively, in the two cases using synchronous Boolean update rules. Thereafter, the steady state logical values (i.e., attractor) of all the 294 nodes in 2560 different input combinations from both the scenarios were identified by using in-built functions available in BooleanNet-1.2.4 and the in house code written in Perl script. However, to present these attractor(s) of each sample in a simplified way, only the steady state binary values of the ten macrophage output molecules (viz. IFN_BETA, IL1_ALPHA, IL1_BETA, IL10, IL12, INOS, IP10, NO, TNF_ALPHA, and c_FOS) were plotted from each attractor(s) state using the network visualization software Gephi (http://gephi.github.io/) and were successively tested for the presence of multiple attractors in the system in uninfected and infected scenarios. On the other hand, the differential activation of the FACTORi in splicing mechanism and its role in the regulation of the network dynamics is further analyzed and discussed in Additional file 1: Text S4 and Additional file 5: Figure S4.

Furthermore, in order to validate our model with experimental data, time-course microarray expression data for the two cells (viz. T cell and APC) were obtained from two separate experiments from the EBI ARRAYEXPRESS database (E-GEOD: 48978 and 42088, for T cell and APC, respectively) [45]. In these microarray experiments, expression profile of activated human T-helper cell (Affymetrix HT HG-U133+ PM Array Plate) and *Leishmania major* infected dendritic cells (Affymetrix HG-U133 Plus 2.0 Gene Chip) were studied at discrete time-points [46, 47]. In our analysis, we only considered the expression values at four time-points, i.e. 0, 2, 4, and 6-h time-points for T-cell and 0, 2, 4, 8-h time-points for dendritic cells. These expression data were then extracted and binarized using the BOOLNET software that employs K-means clustering algorithm [48]. The zeroth hour-binarized data was used to initialize all the nodes of the respective cells, with either ON or OFF depending on whether the protein shows an up-regulation or a down-regulation at the zeroth hour (BooleanNet Software uses TRUE and FALSE for ON and OFF, respectively; Additional file 1: Text S6). The initial values of the *Leishmania* proteins were considered ON in the infected scenario and OFF in the uninfected scenario. The model was then simulated using the synchronous update rule and validated by comparing the expression of the ten APC output molecules in the infected scenario with the binarized time-course microarray data of the APC [46]. However, it should be noted that the experimental data for the expression of NO molecule is considered as proportionate to the expression values of INOS of the microarray data. The model reached its steady state at the 19th time-step in the infected scenario. As a control of the experiment an uninfected scenario was also created. However, to calibrate the four experimental time-points used in microarray data (i.e. 0, 2, 4, and 6 h) with the discrete time points of our simulation results, logical states of the proteins up to 24 discrete time-steps were considered in this analysis (after comparing the steady state values for both the experimental and simulation results). Thus, a 1-h duration of experimental data was associated by three time-steps of the simulations. The temporal expression profile of the ten output molecules were plotted till the 24th step (i.e., 8 h of experimental data). It is to be mentioned here that since the expression of the output proteins is the best reflection of functioning of the entire signaling cascade, the validation of these previously mentioned ten output molecules is assumed to be sufficient to demonstrate the authenticity of the entire model. The T cell model was also validated in a similar way, i.e., by comparing the time-course expression profile of the output protein molecules as obtained in the synchronous simulation with the experimental data [31].

### Model analysis and perturbation studies

The model was simulated asynchronously (until steady state was reached) to make a qualitative analysis of differences in the expression profiles and functional responses of the APC and T cell output molecules in the infected and the uninfected scenarios. The model was iterated 100 times, and the average values of all the simulations at each time-point were plotted for further analysis. This analysis also helped us to monitor the small fluctuations in the expression pattern of the pathway species over time, which occurs due to the stochasticity in the execution of the pathway reactions inside the cell. The asynchronous simulation also ensures that the errors in the synchronous simulations as well as attractor analysis (through selection of independent random samples) are minimized and further presents an average behavior of the entire system over time. In order to unravel the effect of *Leishmania* infection on the entire T cell signaling cascade at the individual protein level, and then to understand the changes at the pathway level, two-tailed Mann-Whitney U test was carried out on the expression of the 163 T cell intermediate and output molecules. This helped us to identify the proteins that get significantly de-regulated during the infection (at 5 % level of significance). Thereafter, the model was used to predict the phenotypic responses (using Eqs. 1, 2, and 3) in various treatment scenarios using several gene knock-in and knock-out experiments created *in silico* by trying different combinations of ON and OFF of the protein molecules using the in-built “boolean2.modify_states” function of the BooleanNet-1.2.4 software [43]. In order to further confirm the robustness of our predicted combinations of immunotherapeutic targets, Boolean attractor analysis was performed for the perturbation scenarios by varying the input molecules of the model (as mentioned earlier). Logical steady states of all the 296 nodes of the model from different scenarios (i.e., uninfected, infected and perturbed) were identified and out of these steady state sequences, only the steady state values of NO, Th1, and Th2 responses were extracted for the comparisons of the effect of different perturbations in the infected scenario. Hence, following this methodology, this study aims to find the effectiveness of our proposed treatment strategies to revert the infected scenario to an infection-free attractor similar to the uninfected scenario.

## Result

### Pathway enrichment analysis

The gene clusters identified from the co-expression networks of the two microarray expression data sets can be considered as the “functional modules” of the gene interaction networks of the *Leishmania major* infected APC and the activated T cell, respectively. A total of 10 and 24 clusters or functional modules are found from the gene co-expression networks of APC and T cell, respectively (Additional file 2: Figure S1 and Additional file 3: Figure S2). Pathway enrichment analyses of the genes found in these clusters have identified various important intracellular signaling pathways (e.g., cytokine-cytokine receptor, toll-like receptor, JAK-STAT, MAPK, mTOR, T cell receptor, calcium signaling, PI3 kinase, interleukin signaling pathways etc.) of two different cells. The complete list of the pathways found to be enriched in this analysis for APC and T-cell are given in Additional file 1: Text S1/Table S1 and Text S2/Table S2, respectively. These enriched pathways, corresponding to the significantly expressed genes of APC and T cell microarray expression data sets, represent the pathways that are influenced by the *Leishmania* pathogen in the APC and in the activated T cell. However, it should be noted that the pathways found to be enriched in this analysis do not to provide the complete understandings of the molecular mechanisms through which the pathogen infect the APCs. Also, we are unable to capture the dynamic interactions of the APC and T cells’ molecules in the *Leishmania* infected scenario. Hence, the reconstructions of the complete inter- and intra-cellular signaling cascades regulating the APC and T cell functions are performed.

### Features of the reconstructed pathway

**Fig. 1:**
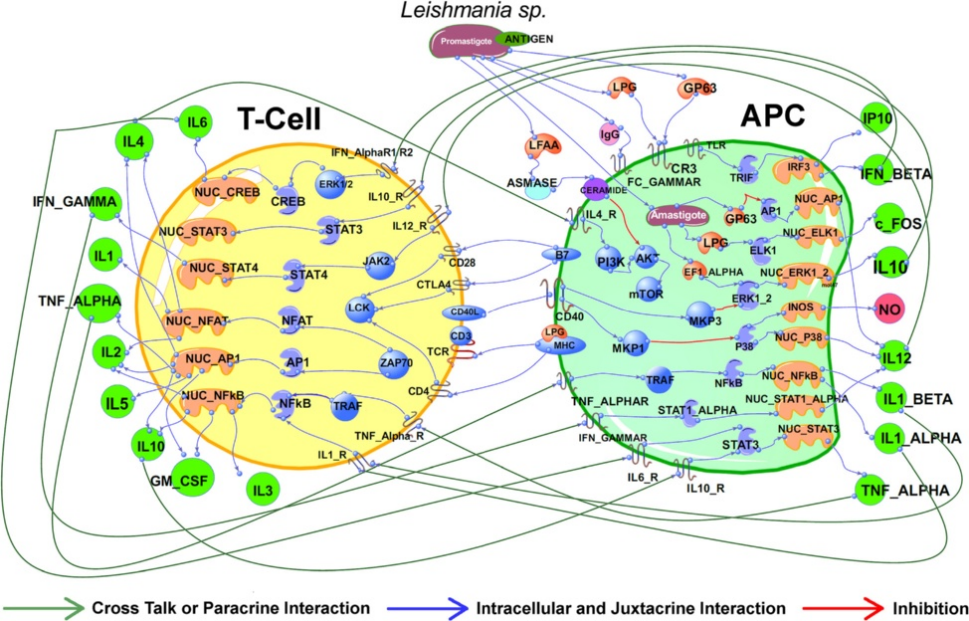
Simplified pathway diagram showing the Leishmania-APC and T cell interaction. The diagram shows the juxtacrine and paracrine regulations between the different cells. The Leishmania antigen molecules are shown in *orange*. The cytoplasmic and nuclear proteins of the APC and T cell are color coded as *blue* and *peach* respectively. The target molecules that are produced as output by the two cells are colored *green* (for protein) and *deep-pink* (for non-protein molecules)

In Fig. 1, a simplified version of the newly reconstructed pathway diagram is presented to provide the brief description of the entire reaction cascade. In this simplified figure, the major inter- and intra-cellular signaling events triggered by important molecules (e.g., MHC, CD40, IL10 etc.) of both the cells and pathogen are provided for the sake of simplicity.

The complete diagram of the newly reconstructed *Leishmania*-APC-T-cell pathway model is provided in Additional file 4: Figure S3. It integrates all possible inter-cellular and intra-cellular signaling events that occur between the two immune cells during *Leishmania* invasion. Here, the interaction of the *Leishmania* molecules, produced from the promastigote and the amastigote forms, with the APC molecules are considered separately. The entire signaling network (i.e. intra- and inter-cellular) consists of a total of 293 nodes, which includes 82 APC molecules, 206 T cell molecules, and 5 *Leishmania*-related molecules, that are involved in more than 400 protein-protein interactions. The intra-cellular signaling cascades considered for modeling the APC and the T cell consists of the major co-receptor signaling pathways, the cytokine pathways, TLR pathways, etc. that play a pivotal role in regulating the outcome of the immune cell’s functional responses. In case of APC, the pathways, which are considered in our model, include the CD40 pathway, the interleukin pathways (viz. IL4, IL6, and IL10), TLR pathways (TLR2, TLR3, TLR4), and the pathways involved in TNF_ALPHA, IFN_GAMMA signaling. Again in T cell, in addition to the core TCR-mediated signaling, seven co-receptor signaling pathways (viz. CD28, CD27, LTBR, CTLA4, ICOS, PD1, and OX40), cytokine pathways (viz. IL1, IL2, IL10, IL12, TNF, and IFN-mediated pathways) and the CRAC channel-mediated calcium pathway are considered.

Various crosstalk reactions are also considered in the model, which depict the bi-directional regulation that exists between the two immune cells. These crosstalk reactions mainly comprise of the juxtacrine signaling events stimulated directly by binding of the co-receptors and the ligand molecules expressed on the T cell and the APC membranes, and the paracrine signaling that are mediated by the diffusible output molecules (mostly cytokines) produced by each cell. Overall 10 crosstalk interactions between the T cell and the APC that effectively regulates the expression pattern of each other are considered. These includes IFN_GAMMA_T, IL4_T, IL6_T, IL10_T, TNF_ALPHA_T molecules secreted from the T cell, and IFN_BETA, TNF_ALPHA, IL12 secreted from the APC that diffuses and activates their corresponding receptor/co-receptors on their neighboring cell to trigger their downstream signaling cascades. The co-receptor ligand molecule interaction considered to be the most important in the model is the one that involves the binding of the CD40 and CD40L_T molecules [3].

The signaling events that begin at the membrane region is then considered to transduce the signal downstream to activate the major signaling pathways, such as, the MAPK, JNK, NFKB, JAK-STAT cascades, which activate a series of transcription factors, that eventually transcribes the output molecules. During *Leishmania* invasion, the antigenic molecules produced by the pathogen activate certain phosphatases (e.g. SHP1, PTP1_B, TCPTP etc.) that interfere with the signaling events of the APC. The antigen molecules considered in the network, such as LPG_L, GP63_L and EF1_Alpha, are shown to have a direct effect on the activities of the ERK1/2 and AP1 transcription factors, the former being upregulated and the latter inhibited or degraded (a detailed description of all the signaling events have been provided in Additional file 1: Text S3).

### Model analysis

#### Attractors

**Fig. 2:**
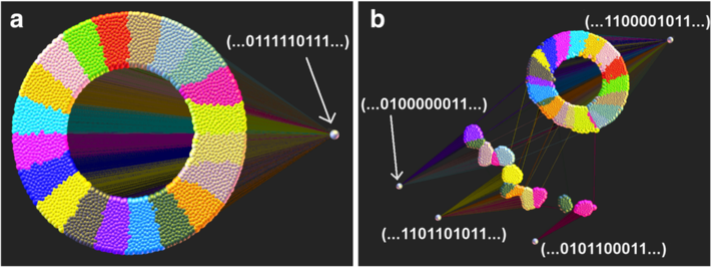
Boolean attractor analysis in uninfected (**a**) and infected scenarios (**b**). The binary values shown in the attractor represents the logical steady state values of 10 macrophage output proteins in the sequence of IFN_BETA, IL1_ALPHA, IL1_BETA, IL10, IL12, INOS, IP10, NO, TNF_ALPHA , and c_FOS, respectively. Different color codes are used to represent the 20 different random samples, and within each sample, 128 nodes represent the input combinations of 7 proteins selected randomly from 51 inputs of the model. In total, there are 2560 combination of initial states denoting the basins of attraction for the entire system

The Boolean attractor analysis performed on 20 independent random samples in the uninfected and the infected scenarios have been plotted in Fig. 2. Here, 128 combinations of input in each of the 20 samples have been grouped together with a specific color code. For simplicity, for the attractor only the sequence of logical states of the molecules in the order of IFN_BETA, IL10, IL12, IL1_ALPHA, IL1_BETA, INOS, IP10, NO, TNF_ALPHA, and c_FOS, is depicted in the network graph. The results of the analysis reveal that given all the FACTORs regulating alternative splicing is assumed to be in ON state, all the 2560 combinations of input (called basins of attractor; each basin is represented as a node in the network graph) in the uninfected scenario, reaches the same Boolean attractor (…0111110111…) (Fig. 2a), while in the infected scenario four different attractors are obtained, viz*.* (…1100001011…), (…0101100011…), (…1101101011…) and (…0100000011…) (Fig. 2b). However, it is to be noted in the infected scenario, 2000 among the 2560 basins (i.e., 78.125 %) reached the (…1100001011…) attractor (including both steady state and cyclic attractor), hereby referred as the major attractor of the system in the infected scenario. These 2000 basins of the major attractor spans all the 20 random samples selected, among which 13 samples exclusively drive to the major attractor, while the remaining 7 samples reach multiple attractors. The (…1101101011…), (…0100000011…), and (…0101100011…) attractors have been attained from 9.375, 9.375, and 3.125 % basins, respectively.

#### Model validation with experimental data

**Fig. 3:**
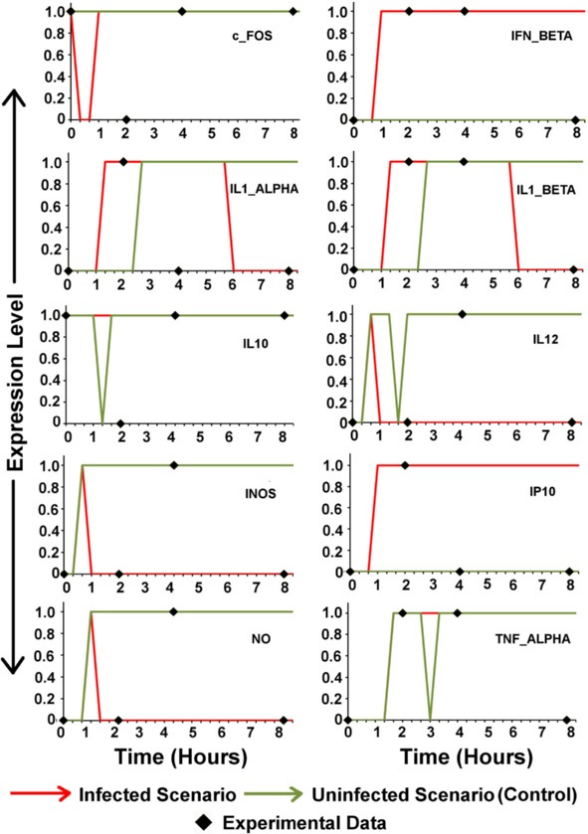
Time-course expression profile of APC output molecules. Expression levels of the output molecules c_FOS, IFN_BETA, IL1_ALPHA, IL1_BETA, IL10, IL12, INOS, IP10, NO, and TNF_ALPHA found in infected, uninfected and experimental conditions. The validation was performed by comparing the expression levels of the infected situations (shown in *red*) with the microarray experimental data (*black diamond*)

The temporal expression profiles of the APC output molecules viz. c_FOS, IL1_ALPHA, IL1_BETA, IFN_BETA, IL10, IL12, IP10, INOS, NO, TNF_ALPHA in the infected (red) and the uninfected scenarios (green) are plotted along-with the binarized microarray data at 0, 2, 4 and 8 h time-points (black diamond) in Fig. 3. This figure depicts that the expression levels of all the 10 output molecules are reaching the steady state values either at 1 (i.e., up-regulation) or 0 (i.e., down-regulation). Here, we observe that expression value of the output molecules at steady state is exactly similar to the value obtained as the major attractor of the system in both the uninfected as well as the infected scenarios (Fig. 2). Qualitative comparison of the expression values reveals that out of these 10 selected output molecules, the steady-state expression value of total 7 molecules viz. c_FOS, IL1_ALPHA, IL1_BETA, IL10, IL12, INOS, and NO in the infected scenario show the exact match with the experimental observations [46]. While c_FOS and IL10 show an expression value of 1 (high expression) in the infected scenario, the other output molecules such as IL1_ALPHA, IL1_BETA, IL12, INOS, and NO have an expression value of 0 (low or no expression) in the infected scenario.

Also, Fig. 3 depicts that at “4 and 8 h” time points, c_FOS and IL10 proteins get upregulated in the simulated infected scenario, which is exactly comparable with the experimentally observed expression levels in microarray data at the same time-points. However, it should be noted that although the expression level of c_FOS protein at “2 h” time point in the simulated infected scenario is not exactly matching with the experimental findings, but the infected model is able to show the downregulation of this protein between the intervals of “0 to 1 h” time points. Both the proteins IL1_ALPHA and IL1_BETA get up regulated at “1 h” time point and subsequently get downregulated at “6 h” time point of the simulated infected scenario. In the experimental data, both of them get upregulated at “2 h” time-point and get downregulated at “4 h” and “8 h” time-points, respectively. In case of IL12, it is observed from Fig. 3 that except a small time interval between 0 and 1 h, this protein remains in the downregulated state throughout the rest of the time-points. The time course microarray data of this protein also shows similar expression level except at “4 h” time-point, in which this protein shows upregulation. Similarly, INOS and NO also show similar expression level at “2 and 8 h” time points as compare to the experimental data. Altogether, the percentage of validation of the simulated *L.major* infected scenario for all the 10 selected proteins at all the three time-points, i.e., 2, 4 and 8 h are 80, 50, and 70 %, respectively.

Also it can be observed that 9 out of 10 output molecules match exactly at least at two time-points. Even though in few cases, the simulation results of the expression values at a particular time point show an apparent mismatch with the experimental observation at that same time-point, but the expression pattern essentially remains the same over time. It can be observed that although the time-course expression of c_FOS from the simulation results appear to be inconsistent with experimental data, i.e., downregulation at 2 h and again upregulation at 4 h time-point, the overall dynamics of the expression essentially remains the same over time, with only a slight deviation of the expression levels (up or down) observed in the respective time-points of experimental and simulation data. Such deviations are also observed in the expression dynamics of IL1_ALPHA, IL12, NO, and INOS molecules. The successful validation of the expression levels of these molecules can be used as valuable indicators of the immune functions of the APC and can be used for fine-tuning of our model to ensure its proper functioning. On the other hand, Fig. 3 also brings out the differences in the expression of the APC output molecules due to the presence of the infection. Here, it is observed that even though the steady state values of the two scenarios (viz. infected and the uninfected) is sometimes similar, as in the cases of c_FOS, IL10, and TNF_ALPHA, the overall temporal expression pattern clearly indicates that the differences are emerging due to the presence of antigen molecules in the model simulation. In the uninfected scenario, the expression of the IL10 and the TNF_ALPHA remains low (in the first few hours) as compared to the infected scenario.

#### Comparison of uninfected and infected scenarios

**Fig. 4:**
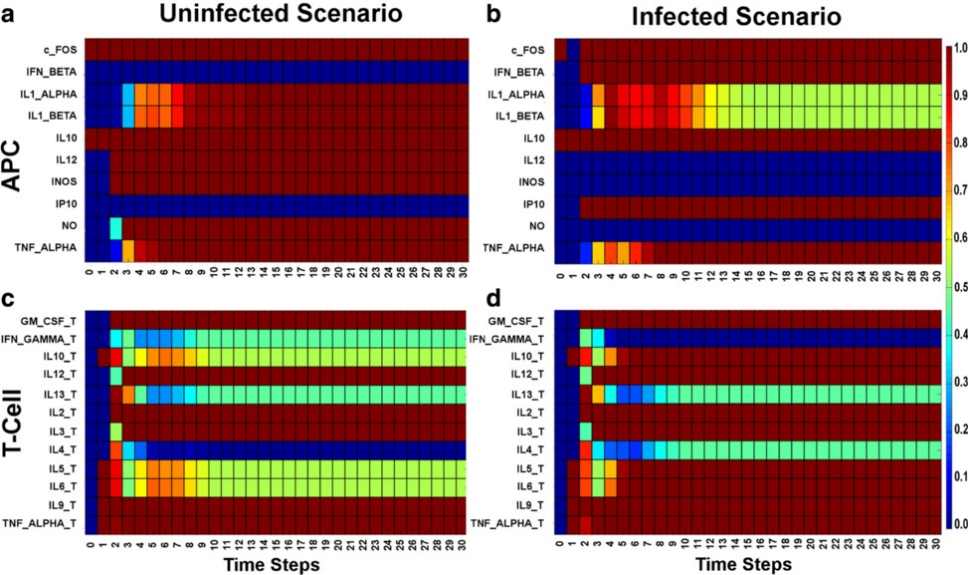
Expression profile of T cell and APC during asynchronous simulation. (**a**) and (**b**) expression of the APC output molecules in the uninfected and infected scenarios, respectively; (**c**) and (**d**) expression of the T cell output proteins in the uninfected and the infected scenarios, respectively

The interference of *Leishmania* proteins in the signaling cascade of APC cell not only modulates the expression of the output molecules and microbicidal activities of APC, but also deregulates the expression of the T cell output molecules by manipulating the normal functioning of T cell activation pathway [49]. Comparing the expression of the APC output proteins in infected and uninfected scenarios (Fig. 4a, b), the simulation results show that invasion of *Leishmania* antigen molecules severely downregulates the expression of IL12, which is a potent T cell stimulator [2, 6]. Simultaneously, the production of INOS and nitric oxide (NO) is also greatly reduced in the infected APC, thereby rendering the cell incapable of performing its microbicidal functions, and creating an immune-suppressed condition, which is favorable for the continued survival of the pathogen inside APC [2, 3]. Besides, in Fig. 4b, the production of IFN_BETA, IP10 (a chemokine) also show an upregulation, indicating an attempt of the APC to eliminate the pathogen from the system [3, 25, 46]. IL1_ALPHA and IL_BETA show minor fluctuations in expression during the infection and slight downregulation [2, 50]. The effect of *Leishmania* infection on the expression pattern of T cell output proteins (Fig. 4c, d) becomes evident from the fact that production of the protective cytokine from the cell, such as IFN_GAMMA_T, is downregulated during the infection, while the productions of interleukins, such as IL10_T, IL4_T, IL5_T, and IL6_T are upregulated, which are mostly implicated as proteins favoring *Leishmania* survival [7, 49, 51, 52]. These results supported by the previous experimental findings also strengthen the validity of our model to a greater extent and enhances its acceptability for further analysis.

#### Effect of infection on T cell signaling cascade

**Fig. 5:**
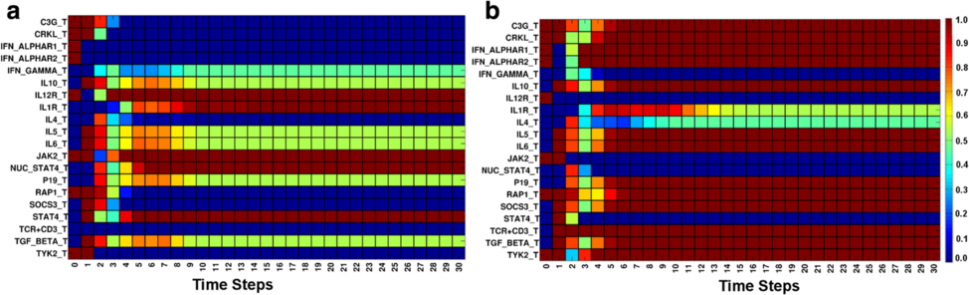
Expression profile of 20 T cell proteins which shows significant deregulation in Mann-Whitney U test. The heat maps depict the protein expression pattern of the T cell signaling proteins under uninfected scenario (**a**) (control); infected scenario (**b**). Significant changes in the expression dynamics are observed for these proteins under these two conditions, which clearly show the effect of Leishmania antigens in the regulation of T cell signaling events

The results of Mann-Whitney U test reveals that out of the expression of 62 proteins in the infected scenario that exhibit a deviation from the uninfected scenario, 20 proteins get significantly deregulated (*P* < 0.05). The temporal expression profiles of these 20 proteins (Fig. 5) show that the *Leishmania* infection causes the significant downregulation of the protective cytokines, such as IFN_GAMMA_T, and enhances the synthesis of TGF_BETA_T, and IL10_T from the T cell, which contributes to the decline in the immune-competency of the T cell and formation of an immune-suppressed condition as observed during *L.major* infections in susceptible patients [5, 6, 53, 54]. It is interesting to note that while the activation of the cytokines, such as IL4_T, IL5_T, IL6_T, and the receptors, IL12R_T [52] and IL1R_T [55], show fluctuations with respect to the control (uninfected scenario), certain other molecules, such as RAP1_T, P19_T, C3G_T, CRKL_T, TYK2_T, and SOC3_T, are distinctly upregulated as a result of the infection. Also, it is observed that the members of the JAK-STAT pathway, such as JAK2_T and STAT4_T are downregulated in the infected scenario (Fig. 5b).

#### Immune response and immunotherapeutic strategies

**Fig. 6:**
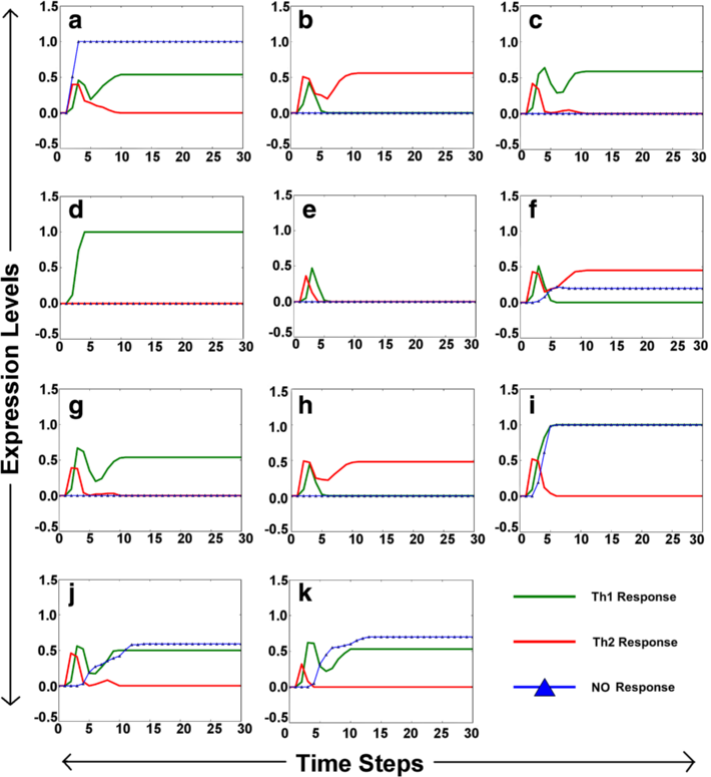
Response dynamics of Th1, Th2, and NO in uninfected, infected, and in different treatment scenarios. **a** uninfected; **b** infected; **c** IL12 [ON]; **d** IFN_GAMMA_T [ON]; **e** MKP_T [ON]; **f** TLR3 [ON]; **g** SHP2_T [OFF]; **h** SHC_T [OFF]; **i** TLR2 [OFF]; **j** TLR3 [ON] and SHP2_T [OFF]; **k** TLR3, MKP_T [ON] and SHC_T [OFF]

The effector molecules produced at the end of the signaling processes in both T cell and APC manifest itself in the form of a change in the phenotypic behaviors of the cell that leads to disease clearance. Through the model, these immune responses of the entire system are simulated using the functions: TH_1_response (Eq. 1), TH_2_response (Eq. 2), and NO_production (Eq. 3)—signifying healing response (green line), non-healing response (red line) and disease clearance (black triangular markers), respectively (Fig. 6). The pathogen load is one of the major factors, which determines the type of immune response that will be elicited during the infection [4]. When the antigens are OFF (i.e., mimicking a situation with low pathogen load, or no infection), the Th1 and the NO responses are higher as compared to the Th2 response (Fig. 6a) [6, 44]. On the contrary, when the antigen molecules are switched ON (i.e., infection is present), a higher Th2-response is obtained (Fig. 6b) [4, 56].

After validating these immune response functions with published literatures, these functions (Eqs. 1, 2, and 3) confirm their acceptability and authenticity to study the effect of the conventional immunotherapeutic strategies in Leishmaniasis (i.e., IL12 and IFN_GAMMA_T), and also to predict some immunostimulatory targets to enhance anti-*Leishmania* immunity (Table 1). Here, at first, we have tried to study the effect of the commonly practiced IL12 (Fig. 6c) and IFN_GAMMA_T (Fig. 6d) treatments and have observed that even though these immunostimulants can enhance the Th1 response and downregulate the Th2 response, they fail to enhance the NO response. Thereafter, through perturbation analysis we have been able to identify three T cell molecules (viz. MKP_T, SHP2_T, and SHC_T) and two APC molecules (viz. TLR3 and TLR2) that may have a positive role in disease clearance. Single in silico mutation study of these molecules reveals that in the MKP_T in silico knock-in scenario (Fig. 6e), even though the Th1 response of the NO response does not increase, the Th2 response gets downregulated as compared to the infected scenario (Fig. 6b). Knock-in mutation of the APC molecule TLR3 gives rise to an increase in NO response, although it has no significant effect on the T cell response (Fig. 6f). In the case of in silico knock-out mutation studies, we have observed that inhibition of SHP2_T leads to upregulation of the Th1 response and downregulation of the Th2 response (Fig. 6g). SHC_T inhibition on the other hand, does not exhibit any significant change in T cell or NO responses as compared to the infected scenario (Fig. 6h). However, if we use a combinatorial therapy by activating the proteins TLR3 while simultaneously inhibiting SHP2_T, we get a better anti-*Leishmania* immune response (combination 1, Fig. 6j). Alternatively, TLR3 knock-in when combined with SHC_T OFF (knock-out) and MKP_T ON (knock-in) can also give rise to a similar effect (combination 2, Fig. 6k). Besides these combinations, interestingly we have also found that if we inhibit only the expression of TLR2 protein in APC, a very high Th1 response is obtained and simultaneously the NO production is also increased drastically (Fig. 6i). A summary of the combinatorial therapeutic strategies and their outcomes as observed from our analysis is provided in Table 1.

**Table 1:** Unique combinations of proteins that can be used as promising immunotherapeutic targets

Knock-in	Knock-out	Th1 response up-regulation	NO increase	Th2 response down-regulation	Anti-Leishmania Immunity^b^	Figure
IL12^a^	–	Yes	No	Yes	No	6c
IFN_GAMMA_T^a^	–	Yes	No	Yes	No	6d
MKP_T	–	No	No	Yes	No	6e
TLR3	–	No	Yes	No	No	6f
–	SHP2_T	Yes	No	Yes	No	6g
–	SHC_T	No	No	No	No	6h
–	TLR2	Yes	Yes	Yes	Yes	6i
TLR3^c^	SHP2_T^c^	Yes	Yes	Yes	Yes	6j
TLR3, MKP_T^d^	SHC_T^d^	Yes	Yes	Yes	Yes	6k

^a^represents previously known and commonly used immunotherapeutic targets; ^b^represents anti-Leishmania immunity that implies to a state when Th1 and NO response is up-regulated and the Th2 response is down-regulated; ^c^represents the proposed Combination 1; ^d^represents the proposed combination 2 treatment strategy

**Fig. 7:**
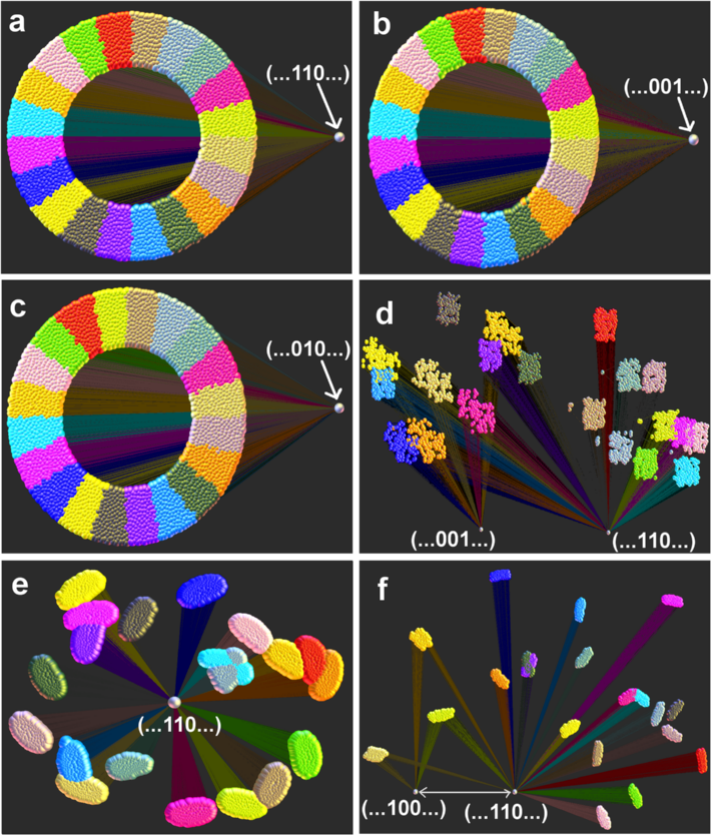
Attractor analysis of the uninfected (**a**), infected (**b**), and in silico treatment scenarios (**c**–**f**). Here, the binary values at the attractor states represent only the logical steady states of NO, Th1, and Th2 responses under uninfected (**a**), infected (**b**), IFN_GAMMA_T [ON] (**c**), TLR2 [OFF] (**d**), TLR3 [ON] and SHP2_T [OFF] (**e**), TLR3, MKP_T [ON] and SHC_T [OFF] (**f**). The logical states of the other nodes/protein molecules are not shown here for the sake of better visualization. The *color codes* are kept same as used in Fig. 2

Further, the results of the Boolean attractor analysis, performed to confirm the robustness of our predictions, reveal that the uninfected and infected scenarios created in our model reaches to unique attractors, viz. (…110…) and (…001…), respectively (Fig. 7 a, b). Here, the attractor denotes the presence/absence of the NO, Th1, and Th2 responses (Fig. 7). The attractor analysis of perturbation studies reveals that the scenario with IFN_GAMMA_T treatment leads to a single attractor (…010…), which is distinct from either the infected or the uninfected attractors (Fig. 7c). However, our predicted targets, viz. TLR2 (Fig. 7d), combination 1 (TLR3 ON and SHP2_T OFF; Fig. 7e), and combination 2 (TLR3, MKP_T ON and SHC_T OFF; Fig. 7f) mostly lead to the infection-free attractor (…110…) similar to the uninfected scenario. Among these, it can be observed that all the 2560 basins in the combination 1 scenario lead only to the infection-free attractor (…110…) (Fig. 7e), while in combination 2, we observe the presence of a bi-stable attractor, oscillating between the (…100…) and (…110…) states (Fig. 7f). TLR2 mutation scenario also shows the presence of two attractors, i.e., (…001…) and (…110…). However, in all these three perturbations the major attractor attained by the system continues to be the desired (…110…) infection-free attractor.

## Discussion

Inadequate knowledge of the complete mechanism of *Leishmani*a invasion inside the host immune system is the key reason for the low success in devising an effective cure to leishmaniasis. In order to overcome this short-coming, it is necessary to gain insight into the precise mechanism of the regulation by which the *Leishmania* antigen molecules takes control of the host cell’s signaling processes. Through this in silico modeling study, we have tried to unravel these regulatory mechanisms by focusing on three important aspects of *Leishmania* immunobiology—(a) effect of *Leishmania* infection on the gene expression or the protein activation pattern in APC and microbicidal activities, (b) effect of the infection on the T cell gene/protein expression pattern at the molecular level and their influence in pathway level to identify the molecular routes by which *Leishmania* inhibits T cell functions, and (c) identification of specific regulators (immunostimulators) that could act as a regulatory switch to skew the Th1/Th2 dynamics towards the healing Th1 response and simultaneously enhance the NO production in order to accelerate the parasite clearance from the host cell.

In this model, we have manually curated the complete signaling cascades of the immune cells depicting the detailed mechanism of regulation of the host protein-protein interaction network by the antigen molecules at various levels of signal transduction and transcriptional activities. Here, we have been able to integrate all the possible routes by which the antigen subverts the host immune responses and modulates the proper functioning of the sentinels of our immune systems, viz. the APCs and the T cells. The model (Additional file 4: Figure S3) depicts the physical binding of the T cell and APC receptors/co-receptors with their corresponding ligands and the subsequent activation mechanism of the downstream proteins in both the cells. The model considers the activation of toll-like-receptor proteins, present in the APC membrane, activate their downstream proteins, which in turns diverges into important signaling routes such as the RAS-RAF mediated MAPK pathway, canonical, and non-canonical NFKB pathway, JAK-STAT pathway, PI3K-PLC Gamma pathway, JNK pathway, etc., and leading to the activation of several transcription factors (e.g., ERK1_2, NFKB, NFAT, AP1, STAT3, etc.) in the nucleus, that in due course, singly or in combination with other transcriptional co-factors initiates the mRNA transcription [39]. These mRNA are then considered to undergo alternative splicing to produce different proteins isoforms with diverse biological functions that regulates the expression of the output molecules. These proteins (principally the cytokines, growth factors, and the cell cycle proteins) synthesized at the end of the cascade, in response to the pathogenic invasion, manifest externally in the form of a change in the cellular behavior, here referred to as a “phenotypic response” viz. the Th1-response, Th2-response, and NO-response (Eqs. 1, 2, and 3) [5].

Boolean attractor analysis reveals the presence of a single attractor in the uninfected scenario and four attractors in the infected scenarios, signifying that depending on the severity of the infection and the presence or absence of certain molecules in the system, *Leishmania* infection may lead the system to multiple levels of infection with varying protein expressions and clinical manifestations (Fig. 2). It can also be observed that the major attractor obtained in these uninfected and infected scenarios matches exactly with the expression values as obtained through our simulations using experimental data in both the scenarios. Asynchronous Boolean simulation is also performed to obtain an average behavior of the entire system under different conditions. Such comparative studies of the infected and uninfected scenarios using asynchronous Boolean simulations brings out the effect of the *Leishmania* infection on the expression of the output molecules in both the APC and the T cell (Figs. 3 and 4), which nicely corroborates with previous experimental studies and strengthens the reliability and authenticity of the model outcomes. We have observed that *Leishmania* infection down-regulates the production of protective cytokines, such as IL12, IL1_ALPHA and IL1_BETA, and microbicidal molecules, such as NO, and simultaneously up-regulating the production of the chemokine, IP10 [3]. The simulation also reveals that in the infected scenario the production of the cytokine IFN_BETA is also upregulated, which is known to have protective functions but only at low doses [25]. The T cell expression profile shows that during *Leishmania* infection, the interleukin molecules *viz.* IL10_T, IL4_T, IL5_T, and IL6_T, gets upregulated, while the expression of IFN_GAMMA_T gets downregulated (Fig. 4c, d). The higher production of the proteins, such as IL10_T and IL4_T and repression of IFN_GAMMA_T synthesis, produces conditions that favor *Leishmania* survival [7], and skews the Th1/Th2 dynamics towards a non-healing response (Fig. 6b) [2, 50].

**Fig. 8:**
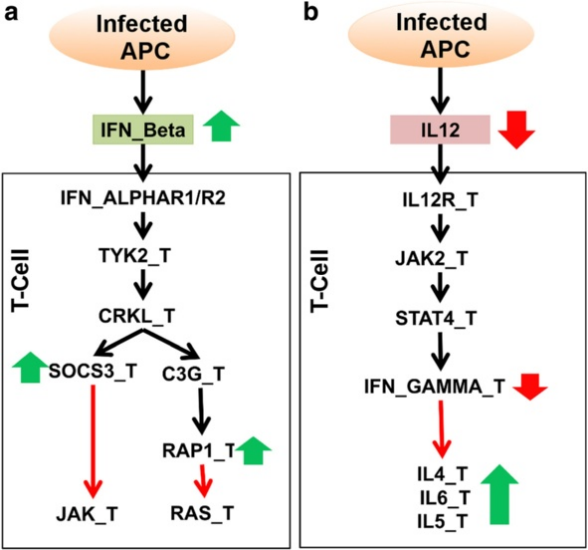
T cell pathways deregulated during leishmaniasis. The schematic diagrams show that the infected APC produces high amount of IFN_Beta, which in turn up-regulates the production of SOCS3 and RAP1 proteins that has negative regulatory effects on its down-stream JAK-STAT and MAPK pathways (**a**); infected APC inhibits the production of the IL12 cytokine which results in upregulation of IL4, IL5, and IL6 cytokine secretion from the T cell by regulating the JAK/STAT and IFN_GAMMA_T protein production (**b**). *Green upward arrow*–protein expression up-regulated; *Red downward arrow*–protein expression down-regulated; *Black arrow*–activation; *Red arrow*–inhibition

A close observation on the results of our Mann-Whitney *U* test analysis (Fig. 5) also predicts some novel and interesting facts about the signaling regulations imposed by the presence of the *Leishmania* infection at the pathway level. Identified from our simulation, this regulatory mechanism of the signaling cascades is presented in Fig. 8. It can be observed that *Leishmania* infection increases the production of the protein IFN_BETA (green upward arrow) and suppresses IL12 (red downward arrow) from the APC. IFN_BETA diffuses and interacts with their corresponding receptors on the T cell thereby enhancing the activation of its downstream TYK2 molecule (black arrow) inside the T cell. Through this analysis, we have tried to determine the possible role of *L.major* infection in modulating the T-cell behavior at the pathway level, and infer that the pathogen upregulates the molecules involved in the TYK-CRKL-C3G pathway. Eventually, it enhances the production of SOCS3 and RAP1 proteins in the T cell (Fig. 8a), two potential negative regulators of JAK-STAT and the RAS-mediated MAPK pathways, respectively (red arrow), which divulges the probable harmful effects of the high levels of IFN_BETA production from the APC that is known to occur during *Leishmania* infection [57, 58]. Moreover, it can be observed that in the T cell (Fig. 8b), the pathogen downregulates the JAK2-STAT4 pathway by inhibiting the synthesis of IL12 cytokine, which results in downregulation of IFN_GAMMA production (red downward arrow) and a consequent increase in the IL4_T, IL5_T, and IL6_T expression (green upward arrow). These findings of the changes occurring at the pathway level have helped us further to identify the key regulators that can act as potential immunostimulators during the infection.

Cytokine therapy is the most widely practiced method of immunotherapy, is employed in the treatment of Leishmaniasis. Immunologists have tried to enhance the expression of IL12 and IFN_GAMMA, the two most potent Th1 response stimulators, which are known to play important role in alleviation of the disease. But, the most common problem faced in such immunotherapies is the inhibitory effect of the IL10 protein, which is overexpressed during the infection that increases the susceptibility to the disease by inhibiting the effects of interferon-gamma treatment and often blocking the synthesis of NO [59], thereby preventing an effective anti-*Leishmania* immunity. In this work, we have tried to simulate the effect of these two immunotherapeutic strategies, viz. IL12 treatment (Fig. 6c) and IFN_GAMMA_T treatment (Fig. 6d), where we have observed that although they are able to enhance the Th1 response and reduce Th2 response, but these strategies fail to induce the NO response, which is necessary to eliminate the disease causing pathogen. Hence, to devise a successful combinatorial immunotherapy, which can bypass the inhibitory effects of immune-suppressive molecules, various molecules that directly or indirectly influence the de-regulated T cell pathways (i.e., JAK2-STAT4 pathway and the TYK2-mediated IFN_BETA pathways) and TLR molecules of the antigen-presenting cell are selectively knocked-in and knocked-out separately and then in combination (Table 1). Thereafter, a set of minimal combinations of protein molecules are identified that could act as regulatory switch to control the Th1/Th2 response and also effectively enhance an anti-*Leishmania* response (Table 1). These molecules include three T cell molecules (viz. SHP2_T, MKP_T and SHC_T), which are also implicated in various cancers and infectious disease treatments, and two APC molecules (viz. TLR2 and TLR3), which are popular targets in many diseases including leishmaniasis [60–63]. A list of antagonists and agonists of these molecules is provided in Additional file 1: Table S4.

Through our study, we suggest that TLR2, which is debated to have the controversial roles in *Leishmania* treatment [23], helps in the parasite survival. This agrees with a recent experimental finding [24], and we propose that TLR2 inhibition can be a useful strategy to up-regulate Th1 and NO response (Fig. 6i). On the other hand it can be understood that TLR3 alone may have a positive role to play in *Leishmania* treatment and may be a positive regulator of NO production (Fig. 6f). It is also interesting to note that although TLR2 inhibition alone is sufficient to drastically enhance the Th1 response and the NO production (Fig. 6i), TLR3 activation requires a synergistic inhibition of the SHP2_T molecule, a phosphatase that inhibits the activity of the JAK-STAT pathway, to gain the desired anti-*Leishmania* response (Fig. 6j). Surprisingly, it is also observed the MAPK phosphatase (MKP_T) when upregulated may inhibit the non-healing Th2 response (Fig. 6e). However, MKP_P and TLR3 upregulation when combined with the inhibition of the adapter molecule SHC_T, a positive regulator of the MAPK cascade, can act as a useful combinatorial target in leishmaniasis treatment (Fig. 6k). Nevertheless to combat leishmaniasis, it may be noted here that since the Th1 subset of helper T cells produces inflammatory cytokines, a constant high Th1 response may often be undesirable in order to avoid harmful side-effects, and hence the two combinations: (1) combination 1: upregulation of TLR3 (i.e. ON state) and downregulation of SHP2_T (i.e., OFF state) and (2) combination 2: upregulations of TLR3, MKP_T, and downregulation of SHC_T, can be considered as better immunotherapeutic strategies than solitary TLR2 inhibition.

The robustness of our predicted combinations was further confirmed through the Boolean attractor analysis, where we observed that the major attractor attained by all the three predicted immunotherapeutic targets resembles with the infection-free attractor (…110…). This is also observed in the uninfected scenario (Fig. 7 d–f), where the NO and Th1 responses are high and the Th2 response is low. In contrast, it can be observed that none of the basins in the IFN_GAMMA_T treatment scenario is able to move the system to this desired (…110…) attractor, which clearly brings out the shortcomings of the conventional immunotherapeutic targets (Fig. 7c). The result of this analysis also highlights the controversial outcomes that may be expected from targeting TLR2 (as mentioned earlier), i.e., TLR2 knock-out may lead to two separate attractors, (…100…) and (…110…). However, it is to be noted that the major attractor obtained in the TLR2 knock-out scenario is the infection-free attractor (…110…), while only a small fraction reaches the attractor (…100…), where although the NO production is high, both the Th1 and the Th2 responses gets downregulated (Fig. 7d). Also, a comparative analysis of the combination 1 and combination 2 scenarios reveals that combination 1 may be considered a better target as compared to the others, as this is the only scenario where we can observe a complete reversal of the infected scenario to a situation (…110… attractor) similar to the uninfected scenario. However, since the combination 2 is leading to a bi-stable attractor, which is oscillating between the major attractor (…110…) and minor attractor (…100…) states, this may also be useful in cases where a constant high NO production is required accompanied with an intermittent up-regulation of Th1 response for patients pre-disposed to inflammatory diseases.

It is important to note that in order to reduce the complexity of the model and due to lack of complete information about the functional regulations of the isoforms in *Leishmania* infected situation, we have only focused on the alternative splicing mechanism at the post-transcriptional level. However, this model may further be extended to study the effect of the alternatively spliced isoforms of the input molecules [64]. For example, TLR3 mRNA molecule is alternatively spliced to produce a smaller 60 kDa isoform, which has been observed to be overexpressed in Glioblastoma cell lines. In future, RNA seq analysis of *Leishmania* infected human APC may provide further insight into the expression of such alternatively spliced isoforms in case of *Leishmania* infection scenario. This may also give a better understanding of the precise regulatory mechanisms underlying the differential protein expression due to the pathogenic invasion.

## Conclusions

The switching between the Th1/Th2 responses during *Leishmania* invasion has important implications in Leishmaniasis treatment, and hence effective regulation of this switching mechanism is important for devising a proper cure for the disease. In this work, we have been able to capture some of the vital aspects of *Leishmania* infection and the mechanism through which the interaction of the *Leishmania* antigen molecules with the APC signaling proteins modulate the microbicidal activity of both the APC and T cell. Although our model does not deal with the dynamics of the entire system due to the large number of unknown parameter sets, but through the logical analysis of the integrated *Leishmania*-APC-T-cell model, we have been able to precisely highlight the inhibitory effects of *Leishmania* infection on the T cell’s signaling routes and Th1/Th2 immune responses. Here, we suggest that *Leishmania* infections enhances the secretion of the IFN_BETA from the APC, which in turn can upregulate the production of the RAP1 and SOCS3 proteins inside the T cell, the potential inhibitors of MAPK and JAK-STAT signaling pathways, respectively, via the TYK2-mediated pathway. The other T cell pathway affected in *Leishmania* infection is the JAK2-STAT4 pathway. Enhancing the activity of this pathway in the T cell by inhibition of the phosphatase SHP2, and simultaneously regulating the activity of the TLR3 molecule in the APC, we have also been able to identify certain unique combinations of proteins, which can act as regulatory switch to shift the Th2 response towards the Th1 response, and at the same time can increase the production of NO. The study highlights a negative role of the T cell SHC molecule and a positive role of the MKP molecule in leishmaniasis treatment. Attractor analysis study firmly establishes the reasons for the failure of the conventional immunotherapeutic targets, such as IFN_GAMMA_T treatment, and ensures that our proposed combinations of protein molecules when targeted reverts the system to an infection-free attractor. Hence, it may be inferred that the proposed combinations of target molecules can be efficiently used as potent immunostimulators to yield an effective anti-*Leishmania* immune response and expedite the process of parasite clearance from the system. We also hope that in future this computational study will be a useful tool for identification of important immune-stimulatory targets for better treatment and alleviation of leishmaniasis.

## Additional files

Additional file 1: **This file contains the following Supplementary Materials.** **Text S1.** Construction of gene co-expression network from *Leishmania* infected APC time course microarray data. **Table S1.** Pathway Enrichment of the significantly expressed genes in the microarray experiment of *Leishmania* infected APC. **Text S2.** Construction of gene co-expression network from time course activated T-cell microarray data. **Table S2.** Pathway Enrichment of the significantly expressed genes in the microarray experiment of activated T-cell. **Text S3.** Brief description of the *Leishmania*-APC-T-cell Signaling Pathways. **Text S4.** Differential regulation of different splicing FACTORS and isoforms. **Table S3.** List of all known alternatively spliced isoforms of the output molecules of both APC and T-cell. **Text S5.** Logical Equations used to model the reaction mechanisms in T-cell and APC during *Leishmania* infection. **Text S6.** Binary initial values of the reaction nodes considered in the Logical equations from binarization of microarray expression data. **Table S4.** List of agonist and antagonist of the proposed targets (DOCX 128 kb)

Additional file 2: Figure S1. Gene clusters identified in *Leishmania major* infected APC microarray data. This figure contains total 10 clusters or functional modules, which have been identified from the gene co-expression network generated from the time course microarray expression data of *Leishmania major* infected APC [EBI-ArrayExpress (ID: E-GEOD-42088)]. The names of the nodes in all the cluster diagrams are assigned according to the probe IDs used in HG-U133_Plus_2 Affymetrix GeneChip for human cell. (TIF 2077 kb)

Additional file 3: Figure S2. Gene clusters identified in active T-cell microarray data. This figure contains total 24 clusters or functional modules, which have been identified from the gene co-expression network generated from the time course microarray expression data of activate T-cell [EBI-ArrayExpress (E-GEOD-48978)]. The node names used in each cluster are in accordance with the probe IDs used in Affymetrix HT_HG-U133_Plus_PM array plate. (TIF 4656 kb)

Additional file 4: Figure S3. Comprehensive diagram of T-cell, APC and *Leishmania* pathogenic protein-protein interaction network. The diagram presents an integrated view of the T-cell and APC interaction signaling pathway during *Leishmania* infection. The different molecules involved in the signaling cascade have been color coded according to its type and cellular location. The molecules colored as red signify the *Leishmania* antigen molecules. The interaction lines have been color coded according to the type of chemical reaction such as phosphorylation (blue), inhibition (red), activation (green) etc. (TIF 5212 kb)

Additional file 5: Figure S4. Attractor analysis of the uninfected and infected scenarios under the differential activation of the splicing factors. (A) In the uninfected scenario the system reach two stable steady state attractors, in which the expressions of IFN_BETA, IL10, IL12, IL1_ALPHA, IL1_BETA, INOS, IP10, NO, TNF_ALPHA and C_FOS proteins are (0111110111) or (0110010111). (B) In the infected scenario, the system reach two stable steady state attractors namely (1100001011) and (1101101011), respectively. (TIF 339 kb)

## Abbreviations

APC: antigen-presenting cell
Th1 response: type I T-helper cell cytokine response
Th2 response: type II T-helper cell cytokine response
NO: nitric oxide molecule
LPG: lipophosphoglycan
PPI: protein-protein interaction
CMI: cell-mediated immunity
