# Supplementary material for: Identification of Th1/Th2 regulatory switch to promote healing response during leishmaniasis: a computational approach
Source: EURASIP J Bioinform Syst Biol. 2015 Dec 1;2015:13. doi: 10.1186/s13637-015-0032-7 (PMC4666900; doi:10.1186/s13637-015-0032-7)
Supplement: Supplementary file 1 — This file contains the following Supplementary Materials. Text S1. Construction of gene co-expression network from Leishmania infected APC time course microarray data. Table S1. Pathway Enrichment of the significantly expressed genes in the microarray experiment of Leishmania infected APC. Text S2. Construction of gene co-expression network from time course activated T-cell microarray data. Table S2. Pathway Enrichment of the significantly expressed genes in the microarray experiment of activated T-cell. Text S3. Brief description of the Leishmania-APC-T-cell Signaling Pathways. Text S4. Differential regulation of different splicing FACTORS and isoforms. Table S3. List of all known alternatively spliced isoforms of the output molecules of both APC and T-cell. Text S5. Logical Equations used to model the reaction mechanisms in T-cell and APC during Leishmania infection. Text S6. Binary initial values of the reaction nodes considered in the Logical equations from binarization of microarray expression data. Table S4. List of agonist and antagonist of the proposed targets (DOCX 128 kb) [file 13637_2015_32_MOESM1_ESM.docx]

**Additional File 1**

**Text S1: Construction of gene co-expression network from *Leishmania* infected APC time course microarray data**

The time course microarray expression data of *Leishmania major* infected human dendritic cell (APC) were taken from the previously performed experiment by Favila *et al.* [[1](#_ENREF_1)]**.** The expression data sets are deposited in GEO (ID: GSE42088) and EBI-ArrayExpress (ID: E-GEOD-42088) database. In that work, the authors have predicted total 849 genes, which are getting significantly expressed after the invasion of *Leishmania* pathogen in human APC. In this analysis, the time course expressions (at 0, 2, 4, 8 and 24 hours) of these 849 probes or genes were extracted and used for the construction of gene co-expression network. In order to do that, at first, the Pearson's correlation coefficients of each pair of genes across the time course microarray data samples were calculated by using the in-built function *corr( )* available in MATLAB R20012. This function also calculates the corresponding *P*-values of the correlation coefficient for each pair of genes in the data set and stores them in the form of a symmetric square matrix. The *P*-value matrix was then used to construct an adjacency matrix (***A***) in which the matrix elements (***A_ij_***) are either **1** (if P-value < 0.01) or **0** (otherwise). The matrix element ***A_ij_*** = 1 signifies that the gene '***i'*** is significantly co-expressed with the gene '***j'*** and there is a connection or undirected arc present between these two genes***.*** In this analysis, total 139,382 arcs are present among 849 significantly expressed genes (or nodes). The possible clusters of genes, formed by at least 3 nodes in the network, are then identified from this huge network using the open source network analysis software: Cytoscape's (version 2.8) GPU enabled App AllegroMCODE (version 2.1) [[2](#_ENREF_2)]. There are total 10 clusters or functional modules identified through this App. The network diagrams of the clusters generated from this analysis are shown in Additional File 2: Figure S1. The names of the nodes in all the cluster diagrams are assigned in the figure according to the probe IDs used in HG-U133_Plus_2 Affymetrix GeneChip for human cell. The genes from each identified functional modules are then used for the further pathway enrichment analysis in bioCompendium (http://biocompendium.embl.de/) web servers. The names of the enriched pathways found for each of the clusters in bioCompendium pathway enrichment web servers are enlisted in Table S1.

**Table S1: Pathway Enrichment of the significantly expressed genes in the microarray experiment of *Leishmania* infected APC**

| **Clusters** | **Pathway Enrichment** |
| --- | --- |
| Cluster 1 | Cytokine-cytokine receptor interaction, Fc gamma R-mediated phagocytosis, Toll-like receptor signaling pathway, MAPK signaling pathway , Jak-STAT signaling pathway, mTOR signaling pathway, Antigen processing and presentation, Chemokine signaling pathway, Apoptosis, Nitrogen metabolism , etc. |
| Cluster 2 | Endocytosis, Toll-like receptor signaling pathway, Fc gamma R-mediated phagocytosis, Cytokine-cytokine receptor interaction, Jak-STAT signaling pathway, MAPK signaling pathway, Chemokine signaling pathway, Spliceosome, etc. |
| Cluster 3 | Fc gamma R-mediated phagocytosis, Phosphatidylinositol signaling system , TGF-beta signaling pathway, Cell cycle, Cell adhesion molecules (CAMs), etc. |
| Cluster 4 | Cytokine-cytokine receptor interaction, Fc gamma R-mediated phagocytosis, MAPK signaling pathway, Phosphatidylinositol signaling system , Spliceosome, Cell cycle, etc. |
| Cluster 5 | Toll-like receptor signaling pathway, Cytosolic DNA-sensing pathway , Jak-STAT signaling pathway, MAPK signaling pathway, mTOR signaling pathway, T cell receptor signaling pathway, Chemokine signaling pathway, etc. |
| Cluster 6 | MAPK signaling pathway, p53 signaling pathway, TGF-beta signaling pathway, Leukocyte trans-endothelial migration , etc. |
| Cluster 7 | Jak-STAT signaling pathway, Phosphatidylinositol signaling system , Fc gamma R-mediated phagocytosis, Chemokine signaling pathway, Antigen processing and presentation, Nitrogen metabolism, Spliceosome , etc. |
| Cluster 8 | Antigen processing and presentation, Cytokine-cytokine receptor interaction, Apoptosis, Ubiquitin mediated proteolysis, etc. |
| Cluster 9 | Phosphatidylinositol signaling system, Chemokine signaling pathway, Leukocyte transendothelial migration, Calcium signaling pathway, Cell adhesion molecules (CAMs), etc. |
| Cluster 10 | Spliceosome |

**Text S2: Construction of gene co-expression network from time course activated T-cell microarray data**

Similar analysis was also performed for activated, time course T-cell microarray expression datasets generated by Zhao et al., and the datasets area available in GEO (ID: GSE48978) and EBI-ArrayExpress (E-GEOD-48978) [[3](#_ENREF_3)]. Their analysis have revealed that in the stimulated state, total 2274 genes get significantly expressed in active T-cells. The co-expression network generated by these significant genes has 551,031 arcs. There are total 24 clusters identified from this huge gene co-expression network, which are depicted in Additional File 3: Figure S2. In the figure, the node names used in each cluster are in accordance with the probe IDs used in Affymetrix HT_HG-U133_Plus_PM array plate. The pathway enrichment analysis of each cluster are performed in GeneCodis [[4](#_ENREF_4)] and the enriched pathways found through this web server are enlisted in Table S2. Only the genes/probes from first 10 clusters (shown in Additional File 3: Figure S2) gave significant enriched pathways, which are enlisted in Table S2.

The significantly enriched pathways, enlisted in Table S1 and Table S2, can be considered as the significantly influenced pathways, which get expressed after the invasion of *Leishmania* pathogen in APC and in the activated T-cell. However, it should be noted that the pathways found to be enriched in this analysis do not provide the complete understandings of the molecular mechanisms through which the *Leishmania* pathogens infect the APCs. It is also unable to describe the dynamic interactions of the secreted proteins/cytokines from both APC and T-cell, and its subsequent effects on the regulation of each other's activities in both uninfected and infected scenarios, respectively. Hence, the reconstructions of the complete inter- and intra cellular signaling cascades, regulating the APC and T-cell functions are very much required.

**Table S2: Pathway Enrichment of the significantly expressed genes in the microarray experiment of activated T-cell**

| **Cluster** | **Pathway Enrichment** |
| --- | --- |
| Cluster 1 | T cell receptor signaling pathway, Calcium signaling pathway, Cytokine-cytokine receptor interaction, Chemokine signaling pathway, Interleukin signaling pathway, Toll-like receptor signaling pathway, MAPK signaling pathway, Ras Pathway, Jak-STAT signaling pathway, PI3 kinase pathway, Antigen processing and presentation, p53 signaling pathway, Cell cycle, Apoptosis, Ubiquitin mediated proteolysis, Spliceosome, etc. |
| Cluster 2 | T cell receptor signaling pathway, Cytokine-cytokine receptor interaction, Chemokine signaling pathway, Jak-STAT signaling pathway, Apoptosis, Spliceosome, etc. |
| Cluster 3 | T cell receptor signaling pathway, T cell activation, MAPK signaling pathway, Chemokine signaling pathway, PI3 kinase pathway, Inflammation mediated by chemokine and cytokine signaling pathway, Apoptosis, etc. |
| Cluster 4 | DNA replication, Tryptophan metabolism, etc. |
| Cluster 5 | Interleukin signaling pathway, Cell cycle, TGF-beta signaling pathway, etc. |
| Cluster 6 | T cell receptor signaling pathway, Interleukin signaling pathway , MAPK signaling pathway, Calcium signaling pathway, Jak-STAT signaling pathway, Ras Pathway, PI3 kinase pathway, Chemokine signaling pathway, Inflammation mediated by chemokine and cytokine signaling pathway, Apoptosis signaling pathway, Spliceosome, etc. |
| Cluster 7 | MAPK signaling pathway, Apoptosis signaling pathway, Leukocyte trans-endothelial migration, Regulation of actin cytoskeleton, etc. |
| Cluster 8 | T cell receptor signaling pathway, Interleukin signaling pathway, Cytokine-cytokine receptor interaction, MAPK signaling pathway, Jak-STAT signaling pathway, Inflammation mediated by chemokine and cytokine signaling pathway, etc. |
| Cluster 9 | Cytokine-cytokine receptor interaction |
| Cluster 10 | Cytokine-cytokine receptor interaction, MAPK signaling pathway, Inflammation mediated by chemokine and cytokine signaling pathway, Leukocyte trans-endothelial migration, Cell adhesion molecules (CAMs), etc. |

**Text S3: Brief description of the *Leishmania*-APC-T-cell Signalling Pathways.**

The pathway enrichment analysis mentioned in the earlier section has given us the insights of the pathways which may get significantly influenced in infected APC and activated T-cell. Hence, the study of the dynamic interplays across the associated molecular species of the significantly enriched pathways of Table S1 and Table S2 would be helpful for the understanding of *Leishmania* invasion mechanisms in APC and followed by the T-cell activation pathways in human host. However, to restrict this work only within the signaling and gene regulation network of the two cells, the other enriched pathways (e.g. metabolic, splicosome etc.) enlisted in Table S1 and Table S2 are not considered for further analyses. Hence, the molecular interactions (e.g. chemical reactions, physical interactions, transcription etc.) of each signaling pathway are reconstructed in this analysis to simulate and analyze the entire mechanisms with the help of logical analysis.

1. ***Leishmania*-APC interaction**

In our reconstructed pathway (Additional File 2: Figure S1), we have considered both the promastiote and the amastigote stages of the parasite. The interaction of the promastigote with the APC occurs at the membrane region where the surface molecules of the pathogen such as GP63_L and LPG_L binds with the complement receptor CR3 of the host cell facilitating the pathogen’s entry [[5](#_ENREF_5),[6](#_ENREF_6)]. The LPG_L molecule of the parasite has also been shown to interact with toll like receptors found on the APC membrane receptors like the TLR2 and TLR4 which are known to phosphorylate the ERK and p38MAP kinases downstream [[7](#_ENREF_7)]. We have also considered the binding of IgG, present on the surface of the opsonized pathogen, to the FC_GAMMAR (i.e the receptor for binding Fc region of the immunoglobulin molecule) on APCs [[8](#_ENREF_8)] which has been shown to stimulate IL10 production by directly activating ERK1/2 [[9](#_ENREF_9)]. During Leishmaniasis, sphingomyelinase ASMASE gets activated in extracellular region, which produces CERAMIDE on the surface of APC [[10](#_ENREF_10)], have also been incorporated here in our model by assuming the presence of a factor LFAA_L (abbreviated for *Leishmania* factor activating ASMASE), produced by the pathogen, that activates ASMASE for the production of CERAMIDE. CERAMIDE once produced, activates PP1 and PP2A both of which are responsible for the inhibition of PKC and de-phosphorylation of AKT in APC cytoplasm [[9](#_ENREF_9)].

A transition line has also been shown in the model indicating the entry of the flagellate motile *Leishmania* promastigote form of the pathogen inside the APC, where it differentiates into a non-motile aflagellate amastiogote form. Inside the cell, the LPG molecule has been shown to interfere with the APC signaling cascade and negatively influence the PKC pathway [[11](#_ENREF_11)]. The LPG molecule that forms a complex with MHC_CLASS_II in the APC is presented to the T cell to cause its activation. The inhibitory effects of GP63_L have also been established in our pathways by showing its inhibitory effects on AP1, NFkB, MARCKS, MRP, c_FOS and mTOR proteins in APC cytoplasm [[12-15](#_ENREF_12)]. GP63_L also activates the phosphatases SHP1, TCPTP and PTP1B as they help in de-phosphorylation of various signaling proteins of APC during *Leishmania* invasion [[16](#_ENREF_16)]. Another *Leishmania* protein that has been included in our model is Elongation factor 1 alpha EF1_ALPHA_L which is also known to activate SHP1 [[17](#_ENREF_17)]. These tyrosine phosphatases (viz. SHP1, TCPTP and PTP1B) de-phosphorylates and mediates deactivation of important downstream transcription factors such as ERK1/2, P38 and JNK [[18-20](#_ENREF_18)] and STAT1_ALPHA [[21-23](#_ENREF_21)].

1. **APC-T-cell interaction**

In order to establish the effect of *Leishmania* infection on the outcome of the T-cell protein expression pattern and the immune responses elicited during the infection, the ligands and the output molecules expressed by the APC have been connected to the T-cells molecules. The APC have been shown to be presenting the *Leishmania* antigen epitope in a processed form denoted as MHC_Class_II:LPG [[24](#_ENREF_24)]. As shown in our model, the TCR: CD3 and MHC_Class_II:LPG interaction triggers the T-cell signal transduction pathways that leads to the activation of the Src family kinases LCK and FYN, and formation of the LAT signalosome [[25-27](#_ENREF_25)]. After this point, the signal cascade branches out into a network of diverse signaling routes, which includes the MAPK pathway, the calcium-mediated NFAT pathway, and the NFKB pathway [[28](#_ENREF_28)]. Upon a pathogenic invasion, the APC expresses a multitude of co-signaling molecules such as B7 and the TNF-like molecules that bind with their corresponding co-receptors on the T-cell membrane to amplify the signal coming from the infected cell and thus causes a sustained T-cell proliferation [[29](#_ENREF_29)]. Here it can be seen that while the B7 molecules (CD80 and CD86) binds to the CD28 co-receptor to activate the SOS:GRB2 mediated MAPK and the PI3K pathways, the TNF-like molecules (TNFRSF9, ICOSL, OX40L, LIGHT) propagates signal to the interior of the cell principally through the TRAF pathway [[29](#_ENREF_29),[30](#_ENREF_30)]. Apart from these juxtacrine signaling at the immunological synapse, we have also considered the CRAC channel that senses the microenvironment of the T-cell for the presence of Ca^2+^ ions and activates the Calcium Pathway. The diffusible cytokines (viz. IL1, IL2, IL10, IL12, TNF alpha and IFN beta) that are produced by the APCs as a result of the pathogen load, and also by the T-cell itself has been shown to further regulate the T-cell proliferation in a paracrine and autocrine fashion respectively [[31-34](#_ENREF_31)].

The pathway also reveals that T-cell and APC interaction has influence on the expression of both the cells. The T-cell regulates the APC through the CD40 pathway which activates MKP1 and MKP3 that further dephosphorylate MAPK proteins P38 and ERK1/2 respectively [[35](#_ENREF_35)]. On the other hand CD40 can also bind with TRAF proteins (TRAF2, TRAF3, TRAF5 and TRAF6) for the activation of MAPK proteins P38 and ERK1/2 [[9](#_ENREF_9),[36](#_ENREF_36)]. Activated T-cell produces effector molecules such as IL10, IL4, IL6, IFN Gamma and TNF alpha, that in-turn controls the signal propagation through the JAK-STAT and TRADD-TRAF pathways of the APC cell [[37-39](#_ENREF_37)].

- - 1. **Output proteins**

The flow of signal from the infected extracellular environment into the cytoplasm and down to the nucleus of these immune cells leads to the activation of specific transcription factors which are responsible for the production of certain effector molecules. These effector molecules that have been included in our model comprises mostly of secreted proteins belonging to the cytokine family, which consists of different interleukins (IL10, IL12, IL1_ALPHA, IL1_BETA, etc.), interferons (IFN_GAMMA, IFN_BETA) and tumour-necrosis factors (TNF_ALPHA) having diverse functions. The other effector molecules produced by the immune cells consists of the growth factors (TGF_BETA), and microbicidal molecules (NO).

**Text S4: Differential regulation of different splicing FACTORS and isoforms**

In order to capture the regulations at the post-transcriptional level, the alternatively spliced isoforms of the T-cell and APC output molecules with known functions have also been included in our model (Additional File 1: Table S3). Total 23 isoforms of 11 genes/proteins [Table S3A(I)] out of 37 output genes/proteins (Table S3), whose functions are known, are considered in the model development. Proteins having only a single functional isoform [enlisted in Table S3B(I)], or having isoforms with unknown functions [listed in Table S3B(II)] have been retained in their original form in the model. The length of all the isoforms have been indicated with first bracket alongside their names. The Principal Isoforms represent the canonical form or the more frequently expressed isoform of the protein, while the alternatively spliced variants are the non-canonical forms, which are relatively rare in occurrence under normal conditions.

**Table S3: List of all known alternatively spliced isoforms of the output molecules of both APC and T-cell.**

1. **Isoforms considered included in our model**
2. **Isoforms with similar function**

|  | **Protein (as used in the model)** | **Principal Isoform (canonical isoform)** | **Alternatively spliced Isoforms (non-canonical isoforms)** | **Functional significance of spliced variants** |
| --- | --- | --- | --- | --- |
|  | PDGF_T | PDGF_AL_T (211) | PDGF_AS_T (196) | PDGF_AL_T and PDGF_AS_T differ in their ability to associate with the extracellular matrix and to bind heparin in vitro. PDGFA_S_T has a lower binding affinity. The overall function remains similar [[40](#_ENREF_40)]. |
|  | TGF_BETA_T | TGFB1_T(390), TGFB2_T(414), TGFB3_T(414) | Several minor isoforms | All TGF Beta isoforms have similar effect on immune cells. Functions of minor isoforms not known [[41](#_ENREF_41)]. |
|  | CYCLIN_D1_T | CYCLIN_D1a_T | CYCLIN_D1b_T | Functions of both the isoforms are similar. Unlike CYCLIN_D1a_T, the non-canonical oncogenic CYCLIN_D1b_T isoform is found only in the nucleus. However CYCLIN_D1b_T is expressed only in cancer derived cell lines [[42](#_ENREF_42)]. |
|  | CYCLIN_D2_T | CYCLIN_D2_iso1_T(289) | CYCLIN_D2_iso2_T(209) | Functions similar. CYCLIN_D2_iso2_T is overexpressed in certain types of cancer [[43](#_ENREF_43)]. |
|  | C_FOS | C_FOS_canonical | C_FOS_2 (169) | C_FOS_2 is degraded at a faster rate than the C_FOS_canonical isoform [[44](#_ENREF_44)]. |
|  | P15_T | P15_138aa_T (138) | P10_T (78) | P15 inhibits cell cycle progression by binding to CDK4 and CDK6 and also via p53 pathway. P10 also inhibits the cell cycle progression, but its function is mediated only via the p53 pathway. P10 does not interact with CDK4 and CDK6 [[45](#_ENREF_45)]. |
|  | PDGFRB_T | PDGFRB_iso1_T (1106) | PDGFRB_iso2_T (336) | Functions similarly [[46](#_ENREF_46)]. |

1. **Isoforms with antagonistic functions**

|  | **Protein (as used in the model)** | **Principal Isoform (canonical isoform)** | **Alternatively spliced Isoforms (non-canonical isoforms)** | **Functional significance of spliced variants** |
| --- | --- | --- | --- | --- |
|  | IL4_T | IL4_long_T (153) | IL4_short_T (137) | The shorter IL4 isoform (IL4delta2) antagonizes the function of the longer canonical IL4 isoform [[47](#_ENREF_47)]. |
|  | IL6_T | IL6_native_T | IL6_Delta4_T | IL6_Delta4_T antagonizes the function of the native IL6 protein [[48](#_ENREF_48)]. |
|  | FASL_T | FASLm_T (281) | FASLs_T (127) | FAS ligand membrane and soluble isoforms has functional differences. The membrane bound form induces apoptosis while the soluble form does not [[49](#_ENREF_49),[50](#_ENREF_50)]. |
|  | BCLX_T | BCLX_L_T (233) | BCLX_S_T (170), BCLX_BETA_T (227) | BCLX_L_T is anti-apoptotic. BCLX_S_T inhibits the function of BCL-2, hence indirectly helps in apoptosis. The function of BCLX_BETA_T is unknown [[51](#_ENREF_51)]. |

1. **Isoforms not included in our model**
2. **Proteins with Single Functional Isoform**

|  | **Protein (as used in the model)** | **Principal Isoform (canonical isoform)** |
| --- | --- | --- |
|  | P19_T | P19_T(166) |
|  | P21_T | P21_T(164) |
|  | HBEGF_T | HBEGF_T(208) |
|  | GM_CSF_T | GM_CSF_T(144) |
|  | IL2_T | IL2_T(153) |
|  | IL3_T | IL3_T(152) |
|  | IFN_GAMMA_T | IFN_GAMMA_T(166) |
|  | IL9_T | IL9_T(144) |
|  | IL10_T | IL10_T(178) |
|  | TNF_ALPHA_T | TNF_ALPHA_T(233) |
|  | IFN_BETA | IFN_BETA(187) |
|  | IL10 | IL10 (178) |
|  | TNF_ALPHA | TNF_ALPHA (233) |
|  | IP10 | IP10(98) |
|  | IL1_ALPHA | IL1_ALPHA (271) |
|  | IL1_BETA | IL1_BETA (269) |
|  | IL5_T | IL5_T(134) |
|  | INOS | iNOS |
|  | CYCLIN_A_T | CYCLIN_A2_T (432) |

1. **Functional Significance of Alternatively Spliced (non-canonical) Isoforms not known**

|  | **Protein (as used in the model)** | **Principal Isoform (canonical isoform)** | **Alternatively spliced Isoforms (non-canonical isoforms)** |
| --- | --- | --- | --- |
|  | BCL2_T | BCL2_ALPHA_T(239) | BCL2_BETA_T(205) |
|  | NUR77_T | NUR77_T(598) | Two other isoforms of length 611aa and 325aa have been identified. |
|  | IL12_T | IL12p40a_T(375); IL12p35_T (328) | IL12p40b_T(330), IL12p40c_T(330); |
|  | P27_T | P27_T(198) | Two other isoforms of length 205aa and 104aa have been identified. |
|  | IL13_T | IL13_T(146) | Another isoform of length 144aa has been isolated |
|  | IL12 | IL12p40a(375); IL12p35(328) | IL12p40b(330), IL12p40c (330); |
|  | CYCLIN_E_T | CYCLIN_E1L_T(410) | CYCLIN_E1S_T(367), CYCLIN_E1_iso3_T(395) |

Here, the expressions of different isoforms from single gene transcript are dependent on the presence of certain cis-regulatory elements and trans-acting factors that has been collectively referred as ‘FACTOR’ in our model. These FACTORs represents specific Spliceosomes responsible for the splice site recognition in each case. However, due to lack of Human cell specific Leishmania major infected RNA seq data of APC, the logical states (activation or inactivation) of the FACTORs determining alternative splicing of the output molecules could not be explicitly determined in Leishmania infected scenario. Hence, in our model these FACTORs were assumed to be ON in all our simulations, signifying that all the alternative isoforms have equal probability of getting expressed. The analysis was performed separately for the uninfected and the infected scenarios, which were created by initializing the Leishmania antigen molecules OFF and ON respectively in the two cases using synchronous Boolean update rules. The analysis was performed separately for the uninfected and the infected scenarios, which were created by initializing the Leishmania antigen molecules OFF and ON respectively in the two cases using synchronous Boolean update rules. The logical equations developed for this purpose are provided in Text S5 and the initial values for uninfected and infected scenarios are provided in Text S6.

However, the concept of attractor analysis was further exploited to understand the differential regulations of the identified 23 FACTORs [*FACTORi where {i=1,2,.....23}* ] associated with the splicing events of 11 output genes. Hence, to observe the effects of the differential activations of all these 23 FACTORS in the production of the corresponding isoforms and the regulation of the dynamics of the entire network, in total 2^23^ combinations of initial states of all the FACTORs have to be considered. Since, finding the attractors of this huge number of input combinations is computationally difficult, hence, to achieve this goal, we have randomly changed the logical states (i.e. 0 or 1) of the 23 FACTORS, and generated 1000 input random samples for both the uninfected and infected scenarios. The inputs files of each of these samples are then used for the simulations and followed by the attractor identifications of uninfected and infected scenarios.

From the simulations of the uninfected and infected scenarios it is observed that both the scenarios are reaching at two stable attractors separately (Additional File 5: Figure S4). It is also observed in the uninfected scenario that the system has reached at two stable steady state attractors, in which the expressions of IFN_BETA, IL10, IL12, IL1_ALPHA, IL1_BETA, INOS, IP10, NO, TNF_ALPHA and C_FOS proteins are either (0111110111) or (0110010111). The first attractor (0111110111) was also found in the simulation in which the probabilities of all the splicing isoforms were taken equally i.e. the initial logical states of all the FACTORs were kept at ON state (Figure 2a). Hence, it can be assumed that the emergence of the second attractor (0110010111) in the simulation is occurring due to the differential expressions and the dynamic interactions of the isoforms in the model. In Additional File 5: Figure S4A, these two attractors nodes (placed at the middle of the circular layouts) are represented by light green and orange colors respectively. The other small nodes, which are connected to these two nodes, are the different samples. Moreover, the subsequent analysis of the logical steady states of NO production, TH1 Response, and TH2 Response are reaching at only one steady state (i.e. 110), which is also accordance with the experimental observations found in the previous studies.

On the other hand, in case of infected scenario, the system is also reaching at two different steady state attractors (1100001011) and (1101101011). Similar to the uninfected scenario, the first attractor (1100001011) is same as the attractor found while keeping all FACTORs at ON state (Figure 2b), and the other attractor (1101101011) is found due to the differential expressions of the different splicing factors in the model. In Additional File 5: Figure S4B, these two attractor nodes are shown by deep green and deep yellow colors at the middle of the circular layout or network. The logical steady states of NO production, TH1 Response, and TH2 Responses observed in the 1000 samples are reaching at two different steady states i.e. either at (001) or (000) and are corresponding to the observed logical steady state attractors (1100001011) and (1101101011), respectively. The logical states of the immune responses (001) are also in accordance with the clinical observations of the *Leishmania* infected APCs. However, the states (000) do not comply with the real biological situation of Leishmania infected immune response patterns. Hence, we have excluded the samples which are driving the systems of the infected scenario towards the second attractor (1101101011) and only take the samples, which are going to the first attractor (1100001011) for further perturbation or drug target identification studies.

**Text S5: Logical Equations used to model the reaction mechanisms in T-cell and APC during *Leishmania* infection.**

1. AKT_T*= (CARMA1_T) *OR* (CDC42+RAC_T) *OR* (COT_T) *OR* (GRB7_T) *OR* (IKK_ALPHA_T *AND* IKK_BETA_T) *OR* (PAK_T) *OR* (PDK1_T) *OR* (PKC_T)
2. AKT*= (IL4R *AND* JAK3 *AND* PI3K *AND* IL4_T)
3. AP1_T*= (  *NOT* IFN_GAMMA_T) *OR* (ATF2_T *AND* C_JUN_T *AND*   *NOT* IFN_GAMMA_T) *OR* (C_FOS_T *AND* C_JUN_T *AND*   *NOT* IFN_GAMMA_T) *OR* (C_JUN_T *AND*   *NOT* IFN_GAMMA_T) *OR* (CRE_T *AND* ATF2_T *AND* C_JUN_T *AND*   *NOT* IFN_GAMMA_T) *OR* (CRE_T *AND* C_JUN_T *AND*   *NOT* IFN_GAMMA_T) *OR* (NUC_ERK1_2_T *AND* C_FOS_T *AND* C_JUN_T *AND*   *NOT* IFN_GAMMA_T) *OR* (NUC_JNK_T *AND* C_FOS_T *AND* C_JUN_T) *OR* (NUC_P38_T *AND* C_FOS_T *AND* C_JUN_T *AND*   *NOT* IFN_GAMMA_T)
4. AP1*= (  *NOT* GP63_L *AND* ERK1_2) *OR* (  *NOT* LPG_L *AND* ERK1_2)
5. ARP2_3_T*= (WASP_T)
6. ASK1_T*= (TRAF2_T)
7. ATF2_T*= (NUC_P38_T)
8. BAD_T*= (  *NOT* AKT_T) *OR* (JNK_T *AND*   *NOT* AKT_T)
9. BAD*= ( *NOT*  AKT)
10. BCL10_T*= (CARMA1_T) *OR* (PKC_THETA_T)
11. BCL2_T*= (  *NOT* JNK_T) *OR* (ETS_T) *OR* (NUC_CREB_T)
12. BCLX_L_T*= (  *NOT* BAD_T *AND* FACTOR20 *OR NOT* FACTOR21) *OR* (  *NOT* JNK_T  *AND* FACTOR20 *OR NOT* FACTOR21) *OR* (ETS_T  *AND* FACTOR20 *OR NOT* FACTOR21) *OR* (NUC_NFKB_T  *AND* FACTOR20 *OR NOT* FACTOR21)
13. BCLX_S_T*= (  *NOT* BAD_T *AND* FACTOR21 *OR NOT* FACTOR20) *OR* (  *NOT* JNK_T  *AND* FACTOR21 *OR NOT* FACTOR20) *OR* (ETS_T  *AND* FACTOR21 *OR NOT* FACTOR20) *OR* (NUC_NFKB_T  *AND* FACTOR21 *OR NOT* FACTOR20)
14. BCLX_T*= (BCLX_L_T *AND NOT* BCLX_S_T) *OR* (BCLX_L_T)
15. C_FOS_2*= (ERK1_2 *AND*   *NOT* GP63_L *AND* FACTOR11 *OR NOT* FACTOR10) *OR* (NUC_STAT3 *AND* FACTOR11 *OR NOT* FACTOR10) *OR* (NUC_ELK1 *AND* FACTOR11 *OR NOT* FACTOR10)
16. C_FOS_CANONICAL*= (ERK1_2 *AND*   *NOT* GP63_L *AND* FACTOR10 *OR NOT* FACTOR11) *OR* (NUC_STAT3 *AND* FACTOR10 *OR NOT* FACTOR11) *OR* (NUC_ELK1 *AND* FACTOR10 *OR NOT* FACTOR11)
17. C_FOS_T*= (C_JUN_T) *OR* (ELK1_T) *OR* (ETS_T) *OR* (NUC_P38_T)
18. C_FOS*= (C_FOS_CANONICAL *OR* C_FOS_2)
19. C_JUN_T*= (C_FOS_T) *OR* (NUC_JNK_T)
20. C3G_T*= (CRKL_T)
21. CABIN1_T*= (  *NOT* CAMK4_T)
22. CALCINEURIN_T*= (  *NOT* CABIN1_T *AND* CAM_T) *OR* (CAM_T *AND*   *NOT* CALCIPRESSIN_T *AND*   *NOT* CABIN1_T)
23. CALCIUM_IN_T*= (CRAC_T *AND* CALCIUM_OUT_T)
24. CAM_T*= (CALCIUM_IN_T) *OR* (VAV_T *AND* CALCIUM_IN_T)
25. CAMK4_T*= (CAM_T)
26. CARMA1_T*= (PKC_THETA_T)
27. CD2_T*= (FYN_T) *OR* (LCK_T)
28. CD3_T*= (LCK_T)
29. CD4_T*= (LCK_T) *OR* (MHC_CLASS_II+LPG_L)
30. CD40*= (CD40L_T)
31. CD8_T*= (LCK_T)
32. CDC42_T*= (PAK_T) *OR* (RAS_T) *OR* (VAV_T)
33. CDC42+RAC_T*= (VAV_T *AND*   *NOT* RAC_GAP_T)
34. CERAMIDE*= (LFAA_L *AND* ASMASE)
35. COT_T*= (RIP1_T) *OR* (TRAF2_T)
36. CR3*= (GP63_L) *OR* (LPG_L)
37. CRAC_T*= (IP3_T)
38. CREB_T*= (RSK_T)
39. CRKL_T*= (TYK2_T)
40. CTLA4_T*=(NUC_NFAT_T)
41. CYC_T*= (  *NOT* AKT_T)
42. CYCLIN_A_T*= (AP1_T *AND*   *NOT* IFN_GAMMA_T) *OR* (NUC_CREB_T) *OR* (NUC_MYC_T)
43. CYCLIN_D1_T*= (CYCLIN_D1A_T *OR* CYCLIN_D1B_T)
44. CYCLIN_D1A_T*= (AP1_T *AND*   *NOT* IFN_GAMMA_T *AND* FACTOR6 *OR NOT* FACTOR7) *OR* (ETS_T *AND* FACTOR6  *OR NOT* FACTOR7) *OR* (NUC_CREB_T *AND* FACTOR6  *OR NOT* FACTOR7) *OR* (NUC_MYC_T *AND* FACTOR6  *OR NOT* FACTOR7) *OR* (NUC_NFKB_T *AND* FACTOR6  *OR NOT* FACTOR7)
45. CYCLIN_D1B_T*= (AP1_T *AND*   *NOT* IFN_GAMMA_T *AND* FACTOR7 *OR NOT* FACTOR6) *OR* (ETS_T *AND* FACTOR7 *OR NOT* FACTOR6) *OR* (NUC_CREB_T *AND* FACTOR7 *OR NOT* FACTOR6) *OR* (NUC_MYC_T *AND* FACTOR7 *OR NOT* FACTOR6) *OR* (NUC_NFKB_T *AND* FACTOR7 *OR NOT* FACTOR6)
46. CYCLIN_D2_ISO1_T*= (NUC_MYC_T *AND* FACTOR8 *OR NOT* FACTOR9)
47. CYCLIN_D2_ISO2_T*= (NUC_MYC_T *AND* FACTOR9 *OR NOT* FACTOR8)
48. CYCLIN_D2_T*= (CYCLIN_D2_ISO1_T *OR* CYCLIN_D2_ISO2_T)
49. CYCLIN_E_T*= (NUC_MYC_T)
50. DAG_T*= (PIP2_T)
51. ELK1_T*= (NUC_ERK1_2_T)
52. ELK1*= (ERK1_2)
53. ERK1_2_T*= (MEK1_2_T *AND*   *NOT* MKP_T)
54. ERK1_2*= (CD40 *AND* MKP3) *OR* (  *NOT* PP2A) *OR* (  *NOT* SHP1 *AND*   *NOT* TCPTP *AND*   *NOT* PTP1B *AND* TLR4 *AND* TRIF) *OR* (  *NOT* SHP1 *AND*   *NOT* TCPTP *AND* TRIF *AND*   *NOT* PTP1B *AND* TLR3) *OR* (LPG_L *AND* TLR2)
55. ETS_T*= (NUC_ERK1_2_T)
56. FASL_T*= (FASLM_T *AND NOT* FASLS_T) *OR* (FASLM_T)
57. FASLM_T*= (ETS_T *AND* FACTOR16 *OR NOT* FACTOR17) *OR* (NUC_NFKB_T *AND* FACTOR16 *OR NOT* FACTOR17)
58. FASLS_T*= (ETS_T *AND* FACTOR17 *OR NOT* FACTOR16) *OR* (NUC_NFKB_T *AND* FACTOR17 *OR NOT* FACTOR16)
59. FKHR_T*= (  *NOT* AKT_T)
60. FYN_T*= (  *NOT* PAG+CSK_T) *OR* (CD45_T *AND*   *NOT* PAG+CSK_T *AND*   *NOT* CBL_T)
61. GAB1_T*= (ERK1_2_T) *OR* (SHC_T)
62. GCKR_T*= (TRAF2_T)
63. GLK_T*= (TRAF2_T)
64. GM_CSF_T*= (NUC_NFKB_T) *OR* (ETS_T *AND* AP1_T)
65. GRB2_T*=PAK_T *OR* SHC_T
66. GRB2+SOS_T*= (CD80 *AND* CD28_T) *OR* (CD86 *AND* CD28_T) *OR* (RAS_GRP_T)
67. GRB7_T*=SHC_T
68. GSK3_BETA_T*= (  *NOT* AKT_T)
69. HBEGF_T*= (ETS_T)
70. HPK1_T*= (LAT_T)
71. IFN_ALPHAR1_T*= IFN_BETA
72. IFN_ALPHAR2_T*= IFN_BETA
73. IFN_BETA*= (TLR3 *AND* TRIF *AND* IRF3) *OR* (TLR4 *AND* TRIF *AND* IRF3)
74. IFN_GAMMA_T*= (NUC_NFAT_T *AND* AP1_T *AND* NUC_STAT4_T) *OR* (  *NOT* IL10)
75. IFN_GAMMAR*= IFN_GAMMA_T
76. IKB_ALPHA_T*= (  *NOT* IKK_ALPHA_T *AND*   *NOT* IKK_BETA_T) *OR* (  *NOT* IKK_BETA_T)
77. IKB_BETA_T*= (  *NOT* IKK_ALPHA_T *AND*   *NOT* IKK_BETA_T *AND*   *NOT* IKK_GAMMA_T)
78. IKK_ALPHA_T*= (NIK_T) *OR* (TRAF2_T)
79. IKK_ALPHA*= (TRADD)
80. IKK_BETA_T*= (BCL10_T) *OR* (IKK_ALPHA_T) *OR* (PKC_THETA_T) *OR* (TRAF2_T)
81. IKK_GAMMA_T*= (BCL10_T *AND* MALT1_T *AND* CARMA1_T) *OR* (CARMA1_T *AND* MALT1_T *AND* BCL10_T *AND* IKK_ALPHA_T *AND* IKK_BETA_T) *OR* (IKK_ALPHA_T *AND* IKK_BETA_T) *OR* (RIP1_T) *OR* (TRAF6_T *AND* MALT1_T)
82. IL1_ALPHA*= (NUC_NFKB)
83. IL1_BETA*= (NUC_NFKB)
84. IL10_T*= (AP1_T *AND* CREB_T *AND*   *NOT* IFN_GAMMA_T) *OR* (NUC_NFAT_T *AND*   *NOT* IFN_GAMMA_T)
85. IL10*= (NUC_ERK1_2)
86. IL10R*= (IL10_T) *OR* (IL10)
87. IL12_T*= (ETS_T *AND* NUC_NFKB_T)
88. IL12*= (  *NOT* MTOR *AND NOT* IL10 *AND* NUC_P38 *AND* NUC_NFKB *AND* NUC_STAT1_ALPHA_P) *OR* (NUC_AP1)
89. IL12R_T*= (IL12)
90. IL12R*= IL12
91. IL13_T*= (NUC_NFAT_T *AND* AP1_T)
92. IL1R_T*= (IL1_BETA)
93. IL2_T*= (AP1_T *AND* NUC_NFAT_T *AND*   *NOT* IFN_GAMMA_T) *OR* (ETS_T *AND* NUC_NFKB_T) *OR* (NUC_NFAT_T *AND* AP1_T) *OR* (NUC_NFKB_T)
94. IL2R_T*=(NUC_NFKB_T)
95. IL3_T*= (ETS_T *AND* NUC_NFKB_T)
96. IL4_LONG_T*= (AP1_T *AND* NUC_NFAT_T *AND*   *NOT* IFN_GAMMA_T *AND* FACTOR12 *OR NOT* FACTOR13)
97. IL4_SHORT_T*= (AP1_T *AND* NUC_NFAT_T *AND*   *NOT* IFN_GAMMA_T *AND* FACTOR13 *OR NOT* FACTOR12)
98. IL4_T*= (IL4_LONG_T *AND NOT* IL4_SHORT_T) *OR* (IL4_LONG_T)
99. IL4R*= IL4_T
100. IL5_T*= (AP1_T *AND*   *NOT* IFN_GAMMA_T)
101. IL6_DELTA4_T*= (AP1_T *AND* CREB_T *AND*   *NOT* IFN_GAMMA_T *AND* FACTOR15 *OR NOT* FACTOR14) *OR* (NUC_NFKB_T *AND* AP1_T *AND* CREB_T *AND*   *NOT* IFN_GAMMA_T *AND* FACTOR15 *OR NOT* FACTOR14)
102. IL6_NATIVE_T*= (AP1_T *AND* CREB_T *AND*   *NOT* IFN_GAMMA_T *AND* FACTOR14 *OR NOT* FACTOR15) *OR* (NUC_NFKB_T *AND* AP1_T *AND* CREB_T *AND*   *NOT* IFN_GAMMA_T *AND* FACTOR14 *OR NOT* FACTOR15)
103. IL6_T*= (IL6_NATIVE_T *AND NOT* IL6_DELTA4_T) *OR* (IL6_NATIVE_T)
104. IL6R*= (IL6_T)
105. IL9_T*= (AP1_T) *OR* (NUC_NFAT_T) *OR* (NUC_NFKB_T)
106. INOS*= (TLR3 *AND* NUC_NFKB *AND* NUC_STAT1_ALPHA_P *AND* P38 *AND* TLR2) *OR* (NUC_AP1) *OR* (  *NOT* IL10)
107. IP10*= (TLR3 *AND* TRIF *AND* IRF3) *OR* (TLR4 *AND* TRIF *AND* IRF3)
108. IP3_T*= (PIP2_T)
109. IRAK1_P*= (IRAK4 *AND* MYD88+TIR+IRAK1)
110. IRF3*= (LPG_L *AND* TLR4 *AND* TRIF) *OR* (TLR3 *AND* TRIF)
111. ITK_T*= (CD2_T) *OR* (LCK_T)
112. JAK1_T*= (  *NOT* SOCS3_T) *OR* (GRB2_T) *OR* (IFN_ALPHAR1_T *AND* IFN_ALPHA_T) *OR* (IFN_ALPHAR1_T *AND* IFN_OMEGA_T) *OR* (IFN_ALPHAR2_T *AND* IFN_ALPHA_T) *OR* (IFN_ALPHAR2_T *AND* IFN_OMEGA_T) *OR* (SHC_T) *OR* (IFN_ALPHAR1_T *AND* IFN_BETA) *OR* (IFN_ALPHAR2_T *AND* IFN_BETA)
113. JAK1*= (IL6_T *AND* IL6R)
114. JAK2_T*= (  *NOT* SHP2_T) *OR* (IL12R_T *AND* IL12)
115. JAK2*= (IFN_GAMMA_T *AND* IFN_GAMMAR) *OR* (IL12 *AND* IL12R)
116. JAK3*= (IL4_T *AND* IL4R)
117. JNK_T*= (MKK_T) *OR* (MKK4_7_T *AND*   *NOT* MKP_T) *OR* (MKK7_T) *OR* (T3JAM_T)
118. JNK*= (  *NOT* SHP1 *AND*   *NOT* TCPTP *AND*   *NOT* PTP1B *AND* IL1_BETA) *OR* (  *NOT* SHP1 *AND*   *NOT* TCPTP *AND*   *NOT* PTP1B *AND* TNF_ALPHA) *OR* (TLR3 *AND* TRIF) *OR* (TLR4 *AND* TRIF)
119. LAT_T*= (ITK_T) *OR* (ZAP70_T)
120. LAT+GRB2+SOS1_T*= (LAT_T *AND* GRB2_T *AND* SOS1_T)
121. LCK_T*= (  *NOT* PAG+CSK_T *AND*   *NOT* LYP_T) *OR* (  *NOT* PAG+CSK_T *AND* CD4_T *AND* MHC_CLASS_II+LPG_L) *OR* (CD4_T *AND* MHC_CLASS_II+LPG_L *AND*   *NOT* PAG+CSK_T *AND*   *NOT* LYP_T) *OR* (CD45_T *AND* CD4_T *AND* MHC_CLASS_II+LPG_L *AND* CD28_T *AND*   *NOT* CBL_T *AND*   *NOT* LYP_T *AND*   *NOT* PAG+CSK_T)
122. LYP_T*= (  *NOT* CSK_T)
123. MALT1_T*= (CARMA1_T) *OR* (PKC_THETA_T)
124. MARCKS*= (  *NOT* GP63_L *AND* PKC)
125. MEF2_T*= (CALCINEURIN_T *AND* P300_T) *OR* (CALCINEURIN_T *AND* P300_T *AND*   *NOT* CABIN1_T *AND*   *NOT* HDAC_T) *OR* (MEF2A_T *AND* MEF2B_T *AND* MEF2C_T *AND* MEF2D_T)
126. MEK1_2_T*= (PAK_T *AND*   *NOT* MKP_T) *OR* (RAF_T *AND*   *NOT* MKP_T) *OR* (RAF1_T *AND*   *NOT* MKP_T)
127. MEKK_T*= (CDC42+RAC_T) *OR* (GCKR_T) *OR* (HPK1_T) *OR* (PAK_T)
128. MEKK1_4_T*= (CDC42+RAC_T) *OR* (RAC1_T)
129. MEKK3_T*= (OSM_T)
130. MEKK4_7_T*= (CDC42+RAC_T)
131. MHC_CLASS_II+LPG_L*= (MHC_CLASS_II *AND* LPG_L)
132. MKK_T*= (ASK1_T) *OR* (MEKK_T)
133. MKK3_6_T*= (MEKK1_4_T) *OR* (MEKK3_T)
134. MKK4_7_T*= (ASK1_T) *OR* (COT_T *AND*   *NOT* MKP_T) *OR* (MEKK4_7_T *AND*   *NOT* MKP_T)
135. MKK7_T*= (MEKK_T) *OR* (TAK1_T)
136. MLK2_T*= (PAK_T)
137. MLK3_T*= (CDC42_T *AND*   *NOT* AKT_T) *OR* (RAC_T)
138. MRP*= (  *NOT* GP63_L *AND* PKC) *OR* (IFN_GAMMAR *AND* IFN_GAMMA_T)
139. MTOR*= (  *NOT* PP1 *AND*   *NOT* PP2A *AND*   *NOT* GP63_L *AND* AKT) *OR* (AKT *AND* IL4R *AND* IL4_T)
140. MYD88*= (LPG_L *AND* TLR4) *OR* (LPG_L *AND* TLR2)
141. MYD88+TIR*= (MYD88 *AND* TIR)
142. MYD88+TIR+IRAK1*= (MYD88+TIR *AND* IRAK1)
143. NCK_T*= (  *NOT* RAS_T) *OR* (PKC_T *AND*   *NOT* RAS_T)
144. NCK+SOS_T*= (NCK_T *AND* SOS_T)
145. NFAT_T*= (CALCINEURIN_T)
146. NFAT+P300+MEF2_T*= (NFAT_T *AND* P300_T *AND* MEF2_T)
147. NFKB_T*= (  *NOT* IKB_BETA_T *AND*   *NOT* IKB_ALPHA_T) *OR* (NIK_T) *OR* (OX40_T *AND* OX40L *AND* PKC_THETA_T *AND* TRAF2_T *AND* RIP1_T *AND* CARMA1_T *AND* MALT1_T *AND* BCL10_T *AND* IKK_ALPHA_T *AND* IKK_BETA_T *AND* IKK_GAMMA_T *AND*   *NOT* IKB_ALPHA_T *AND*   *NOT* IKB_BETA_T) *OR* (TRAF6_T *AND* TAK1_T *AND* IKK_BETA_T *AND*   *NOT* IKB_BETA_T)
148. NFKB*= (ERK1_2 *AND* TLR3 *AND* TRIF) *OR* (ERK1_2 *AND* TLR4 *AND* TRIF *AND NOT* MTOR) *OR* (  *NOT* GP63_L *AND* ERK1_2 *AND* TLR3 *AND* TRIF) *OR* (  *NOT* GP63_L *AND* ERK1_2 *AND* TLR4 *AND* TRIF) *OR* (IRAK1_P *AND* TRAF6) *OR* (TRAF2 *AND* IKK_ALPHA *AND NOT* MTOR)
149. NIK_T*= (COT_T) *OR* (TRAF2_T) *OR* (TRAF5_T)
150. NO*= (INOS)
151. NUC_AP1*= (AP1)
152. NUC_CREB_T*= (CREB_T) *OR* (NUC_ERK1_2_T)
153. NUC_ELK1*= (ELK1)
154. NUC_ERK1_2_T*= (ERK1_2_T)
155. NUC_ERK1_2*= (CD40 *AND* TRAF6 *AND* ERK1_2) *OR* (ERK1_2) *OR* (IGG *AND* FC_GAMMAR *AND* ERK1_2)
156. NUC_JNK_T*= (JNK_T)
157. NUC_MYC_T*=(NUC_ERK1_2_T) *OR* (NUC_NFKB_T)
158. NUC_NFAT_T*= (NFAT_T)
159. NUC_NFKB_T*=(NFKB_T)
160. NUC_NFKB*= (NFKB)
161. NUC_P38_T*=(P38_T)
162. NUC_P38*= (CD40 *AND* TRAF2 *AND* P38) *OR* (CD40 *AND* TRAF3 *AND* P38) *OR* (CD40 *AND* TRAF5 *AND* P38) *OR* (P38)
163. NUC_STAT1_ALPHA_P*= (STAT1_ALPHA_P)
164. NUC_STAT3_T*= (STAT3_T)
165. NUC_STAT3*= (STAT3)
166. NUC_STAT4_T*= (STAT4_T)
167. NUR77_T*= (NFAT+P300+MEF2_T)
168. OSM_T*= (RAC1_T)
169. P10_T*= (NUC_MYC_T  *AND* FACTOR19 *OR NOT* FACTOR18)
170. P15_138AA_T*= (NUC_MYC_T  *AND* FACTOR18 *OR NOT* FACTOR19)
171. P15_T*= (P15_138AA_T *OR* P10_T)
172. P19_T*= (AP1_T *AND* NUC_NFKB_T *AND*   *NOT* IFN_GAMMA_T)
173. P21_T*= (AKT_T) *OR* (NUC_MYC_T)
174. P21RAS_T*=(JAK2_T) *OR* (LAT+GRB2+SOS1_T)
175. P27_T*= (  *NOT* AKT) *OR* (NUC_MYC_T)
176. P38_T*= (MKK3_6_T)
177. P38*= (CD40 *AND* MKP1) *OR* (  *NOT* SHP1 *AND*   *NOT* TCPTP *AND*   *NOT* PTP1B *AND* IL1_BETA) *OR* (  *NOT* SHP1 *AND*   *NOT* TCPTP *AND*   *NOT* PTP1B *AND* TNF_ALPHA) *OR* (LPG_L *AND* TLR4) *OR* (TLR3 *AND* TRIF) *OR* (TLR4 *AND* TRIF)
178. P53_T*=(ETS_T *AND* NUC_P38_T)
179. P70_T*= (PDK1_T)
180. PAG_T*=( *NOT*  CD45_T *AND* LCK_T) *OR* ( *NOT*  CD45_T *AND* FYN_T)
181. PAG+CSK_T*= (PAG_T *AND* CSK_T *AND* FYN_T *AND*   *NOT* CD45_T) *OR* (PAG_T *AND* CSK_T *AND* LCK_T *AND*   *NOT* CD45_T)
182. PAK_T*= (ERK1_2_T *AND*   *NOT* PIP_T) *OR* (GRB2_T) *OR* (NCK_T *AND*   *NOT* PIP_T)
183. PD1_T*=NUC_NFAT_T
184. PDGF_AL_T*= (ETS_T *AND* FACTOR1 *OR NOT* FACTOR2)
185. PDGF_AS_T*= (ETS_T *AND* FACTOR2 *OR NOT* FACTOR1)
186. PDGF_T*= (PDGF_AL_T *OR* PDGF_AS_T)
187. PDGFRB_ISO1_T*= (NUC_MYC_T *AND* FACTOR22 *OR NOT* FACTOR23)
188. PDGFRB_ISO2_T*= (NUC_MYC_T *AND* FACTOR23 *OR NOT* FACTOR22)
189. PDGFRB_T*= (PDGFRB_ISO1_T *OR* PDGFRB_ISO2_T)
190. PDK1_T*= (CARMA1_T) *OR* (PIP3_T)
191. PI3K_T*= (CD80 *AND* CD28_T) *OR* (CD86 *AND* CD28_T) *OR* (GAB1_T) *OR* (GRB2_T) *OR* (ICOSL *AND* ICOS_T) *OR* (RAS_T) *OR* (SHP2_T)
192. PIP2_T*= (PI3K_T) *OR* (PLC_GAMMA_T)
193. PIP3_T*= (PIP2_T) *OR* (PTEN_T)
194. PKC_T*= (JAK1_T)
195. PKC_THETA_T*= (AKT_T) *OR* (DAG_T) *OR* (GLK_T) *OR* (PDK1_T)
196. PKC*= (  *NOT* PP1 *AND*   *NOT* LPG_L) *OR* (  *NOT* PP2A *AND*   *NOT* LPG_L)
197. PLC_GAMMA_T*= (GAB1_T) *OR* (GRB2_T) *OR* (ITK_T) *OR* (LAT_T) *OR* (SHC_T) *OR* (SHP2_T)
198. PP1*= (CERAMIDE)
199. PP2A*= (CERAMIDE)
200. PTP1B*= (GP63_L)
201. RAC_GAP_T*= (DAG_T)
202. RAC_T*= (PAK_T *AND*   *NOT* RAC_GAP_T) *OR* (RAS_T *AND*   *NOT* RAC_GAP_T) *OR* (VAV_T *AND*   *NOT* RAC_GAP_T)
203. RAC1_T*= (NCK_T) *OR* (VAV_T)
204. RAF_T*= (PAK_T) *OR* (PKC_T *AND*   *NOT* AKT_T) *OR* (RAS_T)
205. RAF1_T*= (P21RAS_T)
206. RAP1_T*= (C3G_T)
207. RAS_GAP_T*= (GRB2_T) *OR* (NCK_T)
208. RAS_GRP_T*= (DAG_T *AND* IP3_T) *OR* (LAT_T)
209. RAS_T*= (  *NOT* RAP1_T) *OR* (  *NOT* RAS_GAP_T) *OR* (GRB2+SOS_T) *OR* (GRB7_T *AND*   *NOT* RAS_GAP_T *AND*   *NOT* RAP1_T) *OR* (NCK+SOS_T *AND*   *NOT* RAS_GAP_T *AND*   *NOT* RAP1_T) *OR* (RAS_GRP_T) *OR* (SHC+GRB2+SOS_T *AND*   *NOT* RAS_GAP_T *AND*   *NOT* RAP1_T) *OR* (SHP1+GRB2+SOS_T *AND*   *NOT* RAS_GAP_T *AND*   *NOT* RAP1_T) *OR* (SHP2+GRB2+GAB1+SOS_T *AND*   *NOT* RAS_GAP_T *AND*   *NOT* RAP1_T)
210. RIP1_T*= (TRAF2_T)
211. RSK_T*= (ERK1_2_T)
212. SHC_T*= (GRB7_T) *OR* (IL2_T *AND* IL2R_T) *OR* (PI3K_T) *OR* (PKC_T)
213. SHC+GRB2+SOS_T*= (SHC_T *AND* GRB2_T *AND* SOS_T)
214. SHP1_T*= (  *NOT* ERK1_2_T) *OR* (CD80 *AND* CTLA4_T) *OR* (CD86 *AND* CTLA4_T) *OR* (PDL *AND* PD1_T)
215. SHP1*= (EF1_ALPHA_L) *OR* (GP63_L)
216. SHP1+GRB2+SOS_T*= (SHP1_T *AND* GRB2_T *AND* SOS_T)
217. SHP2_T*= (  *NOT* LCK_T) *OR* (CD80 *AND* CTLA4_T) *OR* (CD86 *AND* CTLA4_T) *OR* (ERK1_2_T) *OR* (SHC_T)
218. SHP2+GRB2+GAB1+SOS_T*= (SHP2_T *AND* GRB2_T *AND* GAB1_T *AND* SOS_T)
219. SLP76_T*= (ITK_T)
220. SOCS3_T*= (CRKL_T) *OR* (NCK_T)
221. SOS1_T*= (ERK1_2_T)
222. STAT1_ALPHA_P*= (IFN_GAMMAR *AND* JAK2 *AND* IFN_GAMMA_T) *OR* (STAT1_ALPHA)
223. STAT1_ALPHA*= (PTP1B *AND* STAT1_ALPHA_P) *OR* (SHP1 *AND* STAT1_ALPHA_P) *OR* (TCPTP *AND* STAT1_ALPHA_P)
224. STAT1_T*= (PKC_T)
225. STAT3_T*= (PKC_T) *OR* (IL10R_T *AND* TYK2_T *AND* IL10)
226. STAT3*= (IL6R *AND* JAK1 *AND* IL6_T) *OR* (IL10R *AND* TYK2 *AND* IL10_T) *OR* (IL12 *AND* IL12R *AND* JAK2)
227. STAT4_T*= (JAK2_T *AND* IL12R_T *AND* IL12) *OR* (JAK2_T)
228. STAT5_T*= (  *NOT* SHP2_T) *OR* (JAK2_T) *OR* (P38_T) *OR* (PAK_T)
229. T3JAM_T*= (TRAF3_T)
230. TAK1_T*= (BCL10_T)
231. TAK1+TAB_T*= (RIP1_T)
232. TCPTP*= (GP63_L)
233. TCR+CD3_T*= (MHC_CLASS_II+LPG_L) *OR* (TCR_T *AND* CD3_T)
234. TGF_BETA_T*= (TGFB1_T *OR* TGFB2_T *OR* TGFB3_T)
235. TGFB1_T*= (AP1_T *AND*   *NOT* IFN_GAMMA_T *AND* FACTOR3 *OR NOT* FACTOR4 *OR NOT* FACTOR5)
236. TGFB2_T*= (AP1_T *AND*   *NOT* IFN_GAMMA_T *AND* FACTOR4 *OR NOT* FACTOR3 *OR NOT* FACTOR5)
237. TGFB3_T*= (AP1_T *AND*   *NOT* IFN_GAMMA_T *AND* FACTOR5 *OR NOT* FACTOR3 *OR NOT* FACTOR4)
238. TLR2*= LPG_L
239. TNF_ALPHA_T*= (AP1_T *AND*   *NOT* IFN_GAMMA_T) *OR* (NUC_STAT3_T)
240. TNF_ALPHA*= (  *NOT* IL10 *AND* NUC_NFKB) *OR* (NUC_STAT3)
241. TNF_ALPHAR_T*= (TNF_ALPHA)
242. TNF_ALPHAR*= (TNF_ALPHA_T)
243. TRADD_T*= (TNF_ALPHA_T *AND* TNF_ALPHAR_T) *OR* (TNF_BETA_T *AND* TNF_ALPHAR_T) *OR* (TNF_ALPHA *AND* TNF_ALPHAR_T)
244. TRADD*= (TNF_ALPHA *AND* TNF_ALPHAR) *OR* (TNF_ALPHA_T *AND* TNF_ALPHAR)
245. TRAF1_T*= (TNFSF9_T *AND* TNFSF9R_T)
246. TRAF2_T*= (TNFSF9_T *AND* TNFSF9R_T)
247. TRAF2*= (TNF_ALPHAR *AND* TRADD *AND* TNF_ALPHA_T) *OR* (OX40L *AND* OX40_T)
248. TRAF3_T*= (CD70 *AND* CD27_T) *OR* (LIGHT *AND* LTBR_T)
249. TRAF3*= (CD40L_T *AND* CD40)
250. TRAF5_T*= (CD70 *AND* CD27_T) *OR* (LIGHT *AND* LTBR_T)
251. TRAF5*= (CD40L_T *AND* CD40)
252. TRAF6_T*= (MALT1_T) *OR* (IL1R_T *AND* IRAK1_T *AND* IRAK4_T *AND* IL1_BETA *AND* MYD88_T)
253. TRAF6*= (CD40L_T *AND* CD40)
254. TRIF*= (LPG_L *AND* TLR4) *OR* (TLR3)
255. TYK2_T*= (IFN_ALPHAR1_T *AND* IFN_ALPHA_T) *OR* (IFN_ALPHAR1_T *AND* IFN_OMEGA_T) *OR* (IFN_ALPHAR2_T *AND* IFN_ALPHA_T) *OR* (IFN_ALPHAR2_T *AND* IFN_OMEGA_T) *OR* (IFN_ALPHAR1_T *AND* IFN_BETA) *OR* (IFN_ALPHAR2_T *AND* IFN_BETA)
256. VAV_T*= (JAK1_T) *OR* (LAT_T *AND* GADS_T *AND* SLP76_T)
257. WASP_T*= (NCK_T)
258. ZAP70_T*= (ABL_T *AND* TCR+CD3_T *AND* MHC_CLASS_II+LPG_L *AND*   *NOT* SHP1_T) *OR* (LCK_T *AND*   *NOT* SHP1_T) *OR* (LCK_T *AND* TCR+CD3_T *AND*   *NOT* LYP_T *AND* FYN_T *AND* ABL_T *AND* VAV_T *AND*   *NOT* SHP1_T *AND* MHC_CLASS_II+LPG_L) *OR* (TCR+CD3_T *AND* MHC_CLASS_II+LPG_L *AND* FYN_T *AND*   *NOT* SHP1)
259. TH_1_RESPONSE*= IL2_T *AND* GM_CSF_T *AND* TNF_ALPHA_T *AND* IFN_GAMMA_T
260. TH_2_RESPONSE*= IL4_T *AND* IL5_T *AND* IL6_T *AND* IL10_T
261. NO_PRODUCTION*=NO

Here, the nodes ***FACTORi {where i=1, 2,.....23}*** are the combinations of the cis and trans-regulatory factors associated with the alternative splicing of the isoforms.

**Text S6: Binary initial values of the reaction nodes considered in the Logical equations from binarization of microarray expression data.**

1. ABL_T=False
2. AKT_T=True
3. AKT=True
4. AP1_T=False
5. AP1=True
6. ARP2_3_T=False
7. ASK1_T=False
8. ASMASE=True
9. ATF2_T=False
10. BAD_T=True
11. BAD=True
12. BCL10_T=False
13. BCL2_T=False
14. BCLX_T=False
15. C_FOS_T=False
16. C_FOS=True
17. C_JUN_T=False
18. C3G_T=True
19. CABIN1_T=True
20. CALCINEURIN_T=False
21. CALCIPRESSIN_T=True
22. CALCIUM_IN_T=False
23. CALCIUM_OUT_T=Random
24. CAM_T=True
25. CAMK4_T=True
26. CARMA1_T=False
27. CBL_T=True
28. CD2_T=False
29. CD27_T=True
30. CD28_T=False
31. CD3_T=True
32. CD4_T=False
33. CD40=False
34. CD40L_T=False
35. CD45_T=True
36. CD70=False
37. CD8_T=False
38. CD80=False
39. CD86=False
40. CDC42_T=False
41. CDC42+RAC_T=False
42. CERAMIDE=True
43. COT_T=False
44. CR3=False
45. CRAC_T=True
46. CRE_T=False
47. CREB_T=False
48. CRKL_T=True
49. CSK_T=True
50. CTLA4_T=False
51. CYC_T=False
52. CYCLIN_A_T=True
53. CYCLIN_D1_T=False
54. CYCLIN_D2_T=False
55. CYCLIN_E_T=True
56. DAG_T=True
57. EF1_ALPHA_L=True
58. ELK1_T=False
59. ELK1=False
60. ERK1_2_T=True
61. ERK1_2=True
62. ETS_T=False
63. FASL_T=False
64. FC_GAMMAR=False
65. FKHR_T=True
66. FYN_T=False
67. GAB1_T=True
68. GADS_T=False
69. GCKR_T=False
70. GLK_T=True
71. GM_CSF_T=False
72. GP63_L=True
73. GRB2_T=False
74. GRB2+SOS_T=False
75. GRB7_T=False
76. GSK3_BETA_T=True
77. HBEGF_T=False
78. HDAC_T=True
79. HPK1_T=True
80. ICOS_T=False
81. ICOSL=False
82. IFN_ALPHA_T=False
83. IFN_ALPHAR1_T=True
84. IFN_ALPHAR2_T=True
85. IFN_BETA=False
86. IFN_GAMMA_T=False
87. IFN_GAMMAR=True
88. IFN_OMEGA_T=True
89. IGG=True
90. IKB_ALPHA_T=False
91. IKB_BETA_T=False
92. IKK_ALPHA_T=False
93. IKK_ALPHA=False
94. IKK_BETA_T=True
95. IKK_GAMMA_T=False
96. IL1_ALPHA=False
97. IL1_BETA=False
98. IL10_T=False
99. IL10=True
100. IL10R_T=True
101. IL10R=False
102. IL12_T=False
103. IL12=False
104. IL12R_T=True
105. IL12R=True
106. IL13_T=False
107. IL1R_T=False
108. IL2_T=False
109. IL2R_T=False
110. IL3_T=False
111. IL4_T=False
112. IL4R=False
113. IL5_T=False
114. IL6_T=False
115. IL6R=False
116. IL9_T=False
117. INOS=False
118. IP10=False
119. IP3_T=False
120. IRAK1_P=True
121. IRAK1_T=False
122. IRAK1=True
123. IRAK4_T=True
124. IRAK4=True
125. IRF3=True
126. ITK_T=False
127. JAK1_T=True
128. JAK1=False
129. JAK2_T=True
130. JAK2=False
131. JAK3=False
132. JNK_T=True
133. JNK=True
134. LAT_T=False
135. LAT+GRB2+SOS1_T=False
136. LCK_T=True
137. LFAA_L=True
138. LIGHT=False
139. LPG_L=True
140. LTBR_T=True
141. LYP_T=False
142. MALT1_T=False
143. MARCKS=False
144. MEF2_T=True
145. MEF2A_T=False
146. MEF2B_T=True
147. MEF2C_T=True
148. MEF2D_T=False
149. MEK1_2_T=True
150. MEKK_T=True
151. MEKK1_4_T=True
152. MEKK3_T=True
153. MEKK4_7_T=True
154. MHC_CLASS_II+LPG_L=True
155. MHC_CLASS_II=True
156. MKK_T=False
157. MKK3_6_T=False
158. MKK4_7_T=False
159. MKK7_T=True
160. MKP_T=False
161. MKP1=True
162. MKP3=True
163. MLK2_T=False
164. MLK3_T=False
165. MRP=False
166. MTOR=False
167. MYD88_T=True
168. MYD88+TIR+IRAK1=False
169. MYD88+TIR=False
170. MYD88=False
171. NCK_T=True
172. NCK+SOS_T=False
173. NFAT_T=False
174. NFAT+P300+MEF2_T=False
175. NFKB_T=False
176. NFKB=False
177. NIK_T=False
178. NO=False
179. NUC_AP1=False
180. NUC_CREB_T=False
181. NUC_ELK1=False
182. NUC_ERK1_2_T=False
183. NUC_ERK1_2=True
184. NUC_JNK_T=False
185. NUC_MYC_T=False
186. NUC_NFAT_T=False
187. NUC_NFKB_T=False
188. NUC_NFKB=False
189. NUC_P38_T=False
190. NUC_P38=False
191. NUC_STAT1_ALPHA_P=False
192. NUC_STAT3_T=False
193. NUC_STAT3=False
194. NUC_STAT4_T=False
195. NUR77_T=False
196. OSM_T=False
197. OX40_T=False
198. OX40L=False
199. P15_T=False
200. P19_T=False
201. P21_T=False
202. P21RAS_T=False
203. P27_T=True
204. P300_T=True
205. P38_T=False
206. P38=False
207. P53_T=False
208. P70_T=False
209. PAG_T=True
210. PAG+CSK_T=False
211. PAK_T=False
212. PD1_T=False
213. PDGF_T=False
214. PDGFRB_T=False
215. PDK1_T=True
216. PDL=False
217. PI3K_T=True
218. PI3K=True
219. PIP_T=False
220. PIP2_T=False
221. PIP3_T=False
222. PKC_T=True
223. PKC_THETA_T=True
224. PKC=True
225. PLC_GAMMA_T=True
226. PP1=False
227. PP2A=False
228. PTEN_T=True
229. PTP1B=False
230. RAC_GAP_T=True
231. RAC_T=False
232. RAC1_T=False
233. RAF_T=True
234. RAF1_T=True
235. RAP1_T=True
236. RAS_GAP_T=False
237. RAS_GRP_T=True
238. RAS_T=False
239. RIP1_T=True
240. RSK_T=True
241. SHC_T=False
242. SHC+GRB2+SOS_T=False
243. SHP1_T=False
244. SHP1+GRB2+SOS_T=False
245. SHP1=True
246. SHP2_T=False
247. SHP2+GRB2+GAB1+SOS_T=False
248. SLP76_T=False
249. SOCS3_T=False
250. SOS_T=True
251. SOS1_T=True
252. STAT1_ALPHA_P=False
253. STAT1_ALPHA=False
254. STAT1_T=False
255. STAT3_T=False
256. STAT3=False
257. STAT4_T=False
258. STAT5_T=False
259. T3JAM_T=True
260. TAK1_T=True
261. TAK1+TAB_T=False
262. TCPTP=True
263. TCR_T=False
264. TCR+CD3_T=False
265. TGF_BETA_T=False
266. TIR=True
267. TLR2=True
268. TLR3=False
269. TLR4=True
270. TNF_ALPHA_T=False
271. TNF_ALPHA=False
272. TNF_ALPHAR_T=False
273. TNF_ALPHAR=True
274. TNF_BETA_T=False
275. TNFSF9_T=False
276. TNFSF9R_T=False
277. TRADD_T=True
278. TRADD=False
279. TRAF1_T=False
280. TRAF2_T=False
281. TRAF2=False
282. TRAF3_T=False
283. TRAF3=False
284. TRAF5_T=False
285. TRAF5=True
286. TRAF6_T=True
287. TRAF6=False
288. TRIF=False
289. TYK2_T=True
290. TYK2=True
291. VAV_T=False
292. WASP_T=False
293. ZAP70_T=False
294. NO_PRODUCTION=False
295. TH_1_RESPONSE=False
296. TH_2_RESPONSE=False
297. FACTOR1= TRUE
298. FACTOR2= TRUE
299. FACTOR3= TRUE
300. FACTOR4= TRUE
301. FACTOR5= TRUE
302. FACTOR6= TRUE
303. FACTOR7= TRUE
304. FACTOR8= TRUE
305. FACTOR9= TRUE
306. FACTOR10= TRUE
307. FACTOR11= TRUE
308. FACTOR12= TRUE
309. FACTOR13= TRUE
310. FACTOR14= TRUE
311. FACTOR15= TRUE
312. FACTOR16= TRUE
313. FACTOR17= TRUE
314. FACTOR18= TRUE
315. FACTOR19= TRUE
316. FACTOR20= TRUE
317. FACTOR21= TRUE
318. FACTOR22= TRUE
319. FACTOR23= TRUE

**Table S4: List of agonist and antagonist of the proposed targets**

| **Targets** | **Antagonist/ Agonist** | **Reference** |
| --- | --- | --- |
| TLR2 | Antagonist- C16H15NO4 | [[52](#_ENREF_52)] |
| TLR3 | Agonist- polyIC_12_U | [[53](#_ENREF_53)] |
| MKP | Agonist- JWH015 | [[54](#_ENREF_54)] |
| SHC | Antagonist- PP2 Inhibitor of Shc/Grb2 interaction- actinomycin D | [[55](#_ENREF_55),[56](#_ENREF_56)] |
| SHP2 | Antagonist- 8-hydroxy-7-(6-sulfonaphthalen-2-yl)diazenyl-quinoline-5-sulfonic acid (NSC-87877) | [[57](#_ENREF_57)] |

**Reference**

1. Favila MA, Geraci NS, Zeng E, Harker B, Condon D, et al. (2014) Human dendritic cells exhibit a pronounced type I IFN signature following Leishmania major infection that is required for IL-12 induction. J Immunol 192: 5863-5872.

2. Smoot ME, Ono K, Ruscheinski J, Wang P-L, Ideker T (2011) Cytoscape 2.8: new features for data integration and network visualization. Bioinformatics 27: 431-432.

3. Zhao S, Fung-Leung WP, Bittner A, Ngo K, Liu X (2014) Comparison of RNA-Seq and microarray in transcriptome profiling of activated T cells. PLoS One 9: e78644.

4. Tabas-Madrid D, Nogales-Cadenas R, Pascual-Montano A (2012) GeneCodis3: a non-redundant and modular enrichment analysis tool for functional genomics. Nucleic acids research 40: W478-W483.

5. Russell DG, Wright S (1988) Complement receptor type 3 (CR3) binds to an Arg-Gly-Asp-containing region of the major surface glycoprotein, gp63, of Leishmania promastigotes. The Journal of experimental medicine 168: 279-292.

6. Talamás-Rohana P, Wright SD, Lennartz MR, Russell DG (1990) Lipophosphoglycan from Leishmania mexicana promastigotes binds to members of the CR3, p150, 95 and LFA-1 family of leukocyte integrins. The Journal of Immunology 144: 4817-4824.

7. Saraiva M, O'Garra A (2010) The regulation of IL-10 production by immune cells. Nat Rev Immunol 10: 170-181.

8. Cunningham AC (2002) Parasitic Adaptive Mechanisms in Infection by Leishmania. Experimental and Molecular Pathology 72: 132-141.

9. Bhardwaj S, Srivastava N, Sudan R, Saha B (2010) Leishmania interferes with host cell signaling to devise a survival strategy. J Biomed Biotechnol 2010: 109189.

10. Majumder S, Dey R, Bhattacharjee S, Rub A, Gupta G, et al. (2012) Leishmania-induced biphasic ceramide generation in macrophages is crucial for uptake and survival of the parasite. Journal of Infectious Diseases 205: 1607-1616.

11. Turco SJ (1999) Adversarial relationship between the Leishmania lipophosphoglycan and protein kinase C of host macrophages. Parasite immunology 21: 597-600.

12. Contreras I, Gomez MA, Nguyen O, Shio MT, McMaster RW, et al. (2010) Leishmania-induced inactivation of the macrophage transcription factor AP-1 is mediated by the parasite metalloprotease GP63. PLoS Pathog 6: e1001148.

13. Corradin S (1999) MARCKS-related Protein (MRP) Is a Substrate for the Leishmania major Surface Protease Leishmanolysin (gp63). Journal of Biological Chemistry 274: 25411-25418.

14. Chawla M, Vishwakarma RA (2003) Alkylacylglycerolipid domain of GPI molecules of Leishmania is responsible for inhibition of PKC-mediated c-fos expression. J Lipid Res 44: 594-600.

15. Jaramillo M, Gomez MA, Larsson O, Shio MT, Topisirovic I, et al. (2011) Leishmania repression of host translation through mTOR cleavage is required for parasite survival and infection. Cell Host Microbe 9: 331-341.

16. Gomez MA, Contreras I, Halle M, Tremblay ML, McMaster RW, et al. (2009) Leishmania GP63 alters host signaling through cleavage-activated protein tyrosine phosphatases. Sci Signal 2: ra58.

17. Nandan D, Yi T, Lopez M, Lai C, Reiner NE (2002) Leishmania EF-1alpha activates the Src homology 2 domain containing tyrosine phosphatase SHP-1 leading to macrophage deactivation. J Biol Chem 277: 50190-50197.

18. Mookerjee Basu J, Mookerjee A, Sen P, Bhaumik S, Sen P, et al. (2006) Sodium antimony gluconate induces generation of reactive oxygen species and nitric oxide via phosphoinositide 3-kinase and mitogen-activated protein kinase activation in Leishmania donovani-infected macrophages. Antimicrob Agents Chemother 50: 1788-1797.

19. Blanchette J, Abu-Dayyeh I, Hassani K, Whitcombe L, Olivier M (2009) Regulation of macrophage nitric oxide production by the protein tyrosine phosphatase Src homology 2 domain phosphotyrosine phosphatase 1 (SHP-1). Immunology 127: 123-133.

20. Halle M, Gomez MA, Stuible M, Shimizu H, McMaster WR, et al. (2009) The Leishmania surface protease GP63 cleaves multiple intracellular proteins and actively participates in p38 mitogen-activated protein kinase inactivation. J Biol Chem 284: 6893-6908.

21. Forget G, Gregory DJ, Whitcombe LA, Olivier M (2006) Role of host protein tyrosine phosphatase SHP-1 in Leishmania donovani-induced inhibition of nitric oxide production. Infect Immun 74: 6272-6279.

22. Zhu W, Mustelin T, David M (2002) Arginine methylation of STAT1 regulates its dephosphorylation by T cell protein tyrosine phosphatase. J Biol Chem 277: 35787-35790.

23. Xu H, An H, Hou J, Han C, Wang P, et al. (2008) Phosphatase PTP1B negatively regulates MyD88- and TRIF-dependent proinflammatory cytokine and type I interferon production in TLR-triggered macrophages. Molecular Immunology 45: 3545-3552.

24. Awasthi A, Mathur RK, Saha B (2004) Immune response to Leishmania infection. Indian Journal of Medical Research 119: 238-258.

25. Moll H, Röllinghoff M (1991) T-cell reactivity to purified lipophosphoglycan from Leishmania major: A model for analysis of the cellular immune response to microbial carbohydrates. Behring Inst Mitt 88: 161-169.

26. Brownlie RJ, Zamoyska R (2013) T cell receptor signalling networks: branched, diversified and bounded. Nature Reviews Immunology 13: 257-269.

27. Kaye P, Scott P (2011) Leishmaniasis: complexity at the host–pathogen interface. Nat Rev Micro 9: 604-615.

28. Yamagishi M, Watanabe T (2012) New Paradigm of T cell Signaling: Learning from Malignancies. J Clin Cell Immunol S 12: 2.

29. Chen L, Flies DB (2013) Molecular mechanisms of T cell co-stimulation and co-inhibition. Nature Reviews Immunology 13: 227-242.

30. Fathman CG, Lineberry NB (2007) Molecular mechanisms of CD4+ T-cell anergy. Nat Rev Immunol 7: 599-609.

31. Akdis M, Burgler S, Crameri R, Eiwegger T, Fujita H, et al. (2011) Interleukins, from 1 to 37, and interferon-gamma: receptors, functions, and roles in diseases. J Allergy Clin Immunol 127: 701-721 e701-770.

32. Bak RO, Mikkelsen JG (2010) Review Regulation of cytokines by small RNAs during skin inflammation.

33. Feske S (2013) Ca(2+) influx in T cells: how many ca(2+) channels? Front Immunol 4: 99.

34. Qu B, Al-Ansary D, Kummerow C, Hoth M, Schwarz EC (2011) ORAI-mediated calcium influx in T cell proliferation, apoptosis and tolerance. Cell Calcium 50: 261-269.

35. Srivastava N, Sudan R, Saha B (2011) CD40-modulated dual-specificity phosphatases MAPK phosphatase (MKP)-1 and MKP-3 reciprocally regulate Leishmania major infection. J Immunol 186: 5863-5872.

36. Rub A, Dey R, Jadhav M, Kamat R, Chakkaramakkil S, et al. (2009) Cholesterol depletion associated with Leishmania major infection alters macrophage CD40 signalosome composition and effector function. Nat Immunol 10: 273-280.

37. Liu D, Uzonna JE (2012) The early interaction of Leishmania with macrophages and dendritic cells and its influence on the host immune response. Front Cell Infect Microbiol 2: 83.

38. Olivier M, Gregory DJ, Forget G (2005) Subversion mechanisms by which Leishmania parasites can escape the host immune response: a signaling point of view. Clin Microbiol Rev 18: 293-305.

39. Kemp K (2000) Cytokine-producing T cell subsets in human leishmaniasis. ARCHIVUM IMMUNOLOGIAE ET THERAPIAE EXPERIMENTALIS-ENGLISH EDITION- 48: 173-176.

40. Pollock RA, Richardson WD (1992) The alternative-splice isoforms of the PDGF A-chain differ in their ability to associate with the extracellular matrix and to bind heparin in vitro. Growth Factors 7: 267-277.

41. Gorelik L, Flavell RA (2002) Transforming growth factor-beta in T-cell biology. Nat Rev Immunol 2: 46-53.

42. Lu F, Gladden AB, Diehl JA (2003) An alternatively spliced cyclin D1 isoform, cyclin D1b, is a nuclear oncogene. Cancer research 63: 7056-7061.

43. Denicourt C, Legault P, McNabb FA, Rassart E (2008) Human and mouse cyclin D2 splice variants: transforming activity and subcellular localization. Oncogene 27: 1253-1262.

44. Jurado J, Fuentes-Almagro CA, Prieto-Alamo MJ, Pueyo C (2007) Alternative splicing of c-fos pre-mRNA: contribution of the rates of synthesis and degradation to the copy number of each transcript isoform and detection of a truncated c-Fos immunoreactive species. BMC Mol Biol 8: 83.

45. de Castro IP, Benet M, Jiménez M, Alzabin S, Malumbres M, et al. (2005) Mouse p10, an alternative spliced form of p15INK4b, inhibits cell cycle progression and malignant transformation. Cancer research 65: 3249-3256.

46. Jin P, Zhang J, Sumariwalla PF, Ni I, Jorgensen B, et al. (2008) Novel splice variants derived from the receptor tyrosine kinase superfamily are potential therapeutics for rheumatoid arthritis. Arthritis Res Ther 10: R73.

47. Arinobu Y, Atamas SP, Otsuka T, Niiro H, Yamaoka K, et al. (1999) Antagonistic effects of an alternative splice variant of human IL-4, IL-4δ2, on IL-4 activities in human monocytes and B cells. Cellular immunology 191: 161-167.

48. Bihl MP, Heinimann K, Rudiger JJ, Eickelberg O, Perruchoud AP, et al. (2002) Identification of a novel IL-6 isoform binding to the endogenous IL-6 receptor. American journal of respiratory cell and molecular biology 27: 48-56.

49. Hohlbaum AM, Moe S, Marshak-Rothstein A (2000) Opposing effects of transmembrane and soluble Fas ligand expression on inflammation and tumor cell survival. The Journal of experimental medicine 191: 1209-1220.

50. Ayroldi E, D’Adamio F, Zollo O, Agostini M, Moraca R, et al. (1999) Cloning and expression of a short Fas ligand: a new alternatively spliced product of the mouse Fas ligand gene. Blood 94: 3456-3467.

51. Boise LH, González-García M, Postema CE, Ding L, Lindsten T, et al. (1993) bcl-x, a bcl-2-related gene that functions as a dominant regulator of apoptotic cell death. cell 74: 597-608.

52. Mistry P, Laird MH, Schwarz RS, Greene S, Dyson T, et al. (2015) Inhibition of TLR2 signaling by small molecule inhibitors targeting a pocket within the TLR2 TIR domain. Proc Natl Acad Sci U S A 112: 5455-5460.

53. Nicodemus CF, Berek JS (2010) TLR3 agonists as immunotherapeutic agents. Immunotherapy 2: 137.

54. Romero-Sandoval EA, Horvath R, Landry RP, DeLeo JA (2009) Cannabinoid receptor type 2 activation induces a microglial anti-inflammatory phenotype and reduces migration via MKP induction and ERK dephosphorylation. Mol Pain 5: 25.

55. Brown JE, Zeiger SL, Hettinger JC, Brooks JD, Holt B, et al. (2010) Essential role of the redox-sensitive kinase p66shc in determining energetic and oxidative status and cell fate in neuronal preconditioning. J Neurosci 30: 5242-5252.

56. Kim HK, Jeong MJ, Kong MY, Han MY, Son KH, et al. (2005) Inhibition of Shc/Grb2 protein-protein interaction suppresses growth of B104-1-1 tumors xenografted in nude mice. Life Sci 78: 321-328.

57. Chen L, Sung SS, Yip ML, Lawrence HR, Ren Y, et al. (2006) Discovery of a novel shp2 protein tyrosine phosphatase inhibitor. Mol Pharmacol 70: 562-570.
