# Supplementary figures and images for: Identification of Th1/Th2 regulatory switch to promote healing response during leishmaniasis: a computational approach
Source: EURASIP J Bioinform Syst Biol. 2015 Dec 1;2015:13. doi: 10.1186/s13637-015-0032-7 (PMC4666900; doi:10.1186/s13637-015-0032-7)

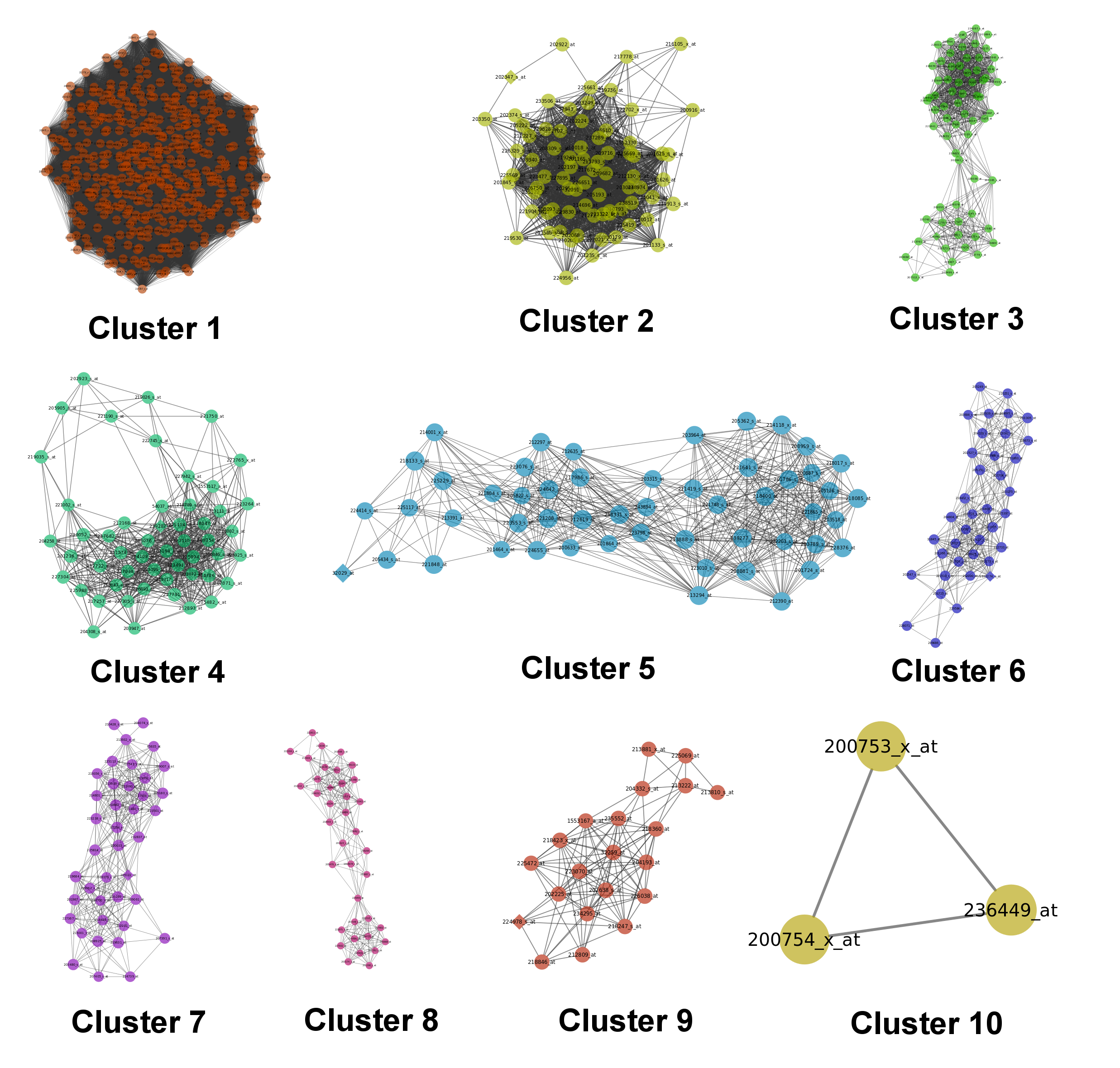

Supplement: Supplementary file 2 — Gene clusters identified in Leishmania major infected APC microarray data. This figure contains total 10 clusters or functional modules, which have been identified from the gene co-expression network generated from the time course microarray expression data of Leishmania major infected APC [EBI-ArrayExpress (ID: E-GEOD-42088)]. The names of the nodes in all the cluster diagrams are assigned according to the probe IDs used in HG-U133_Plus_2 Affymetrix GeneChip for human cell. (TIF 2077 kb) [file 13637_2015_32_MOESM2_ESM.tif]

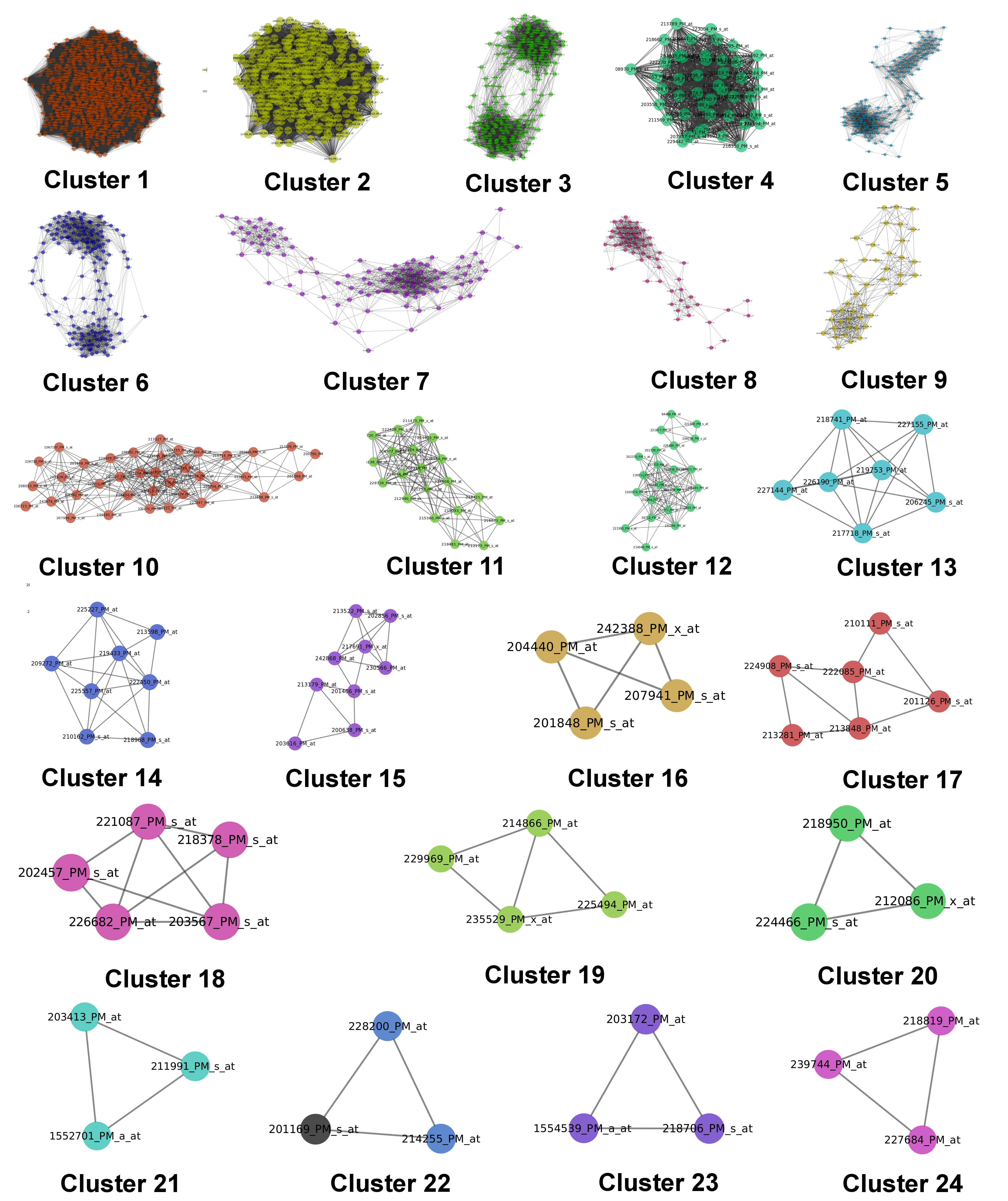

Supplement: Supplementary file 3 — Gene clusters identified in active T-cell microarray data. This figure contains total 24 clusters or functional modules, which have been identified from the gene co-expression network generated from the time course microarray expression data of activate T-cell [EBI-ArrayExpress (E-GEOD-48978)]. The node names used in each cluster are in accordance with the probe IDs used in Affymetrix HT_HG-U133_Plus_PM array plate. (TIF 4656 kb) [file 13637_2015_32_MOESM3_ESM.tif]

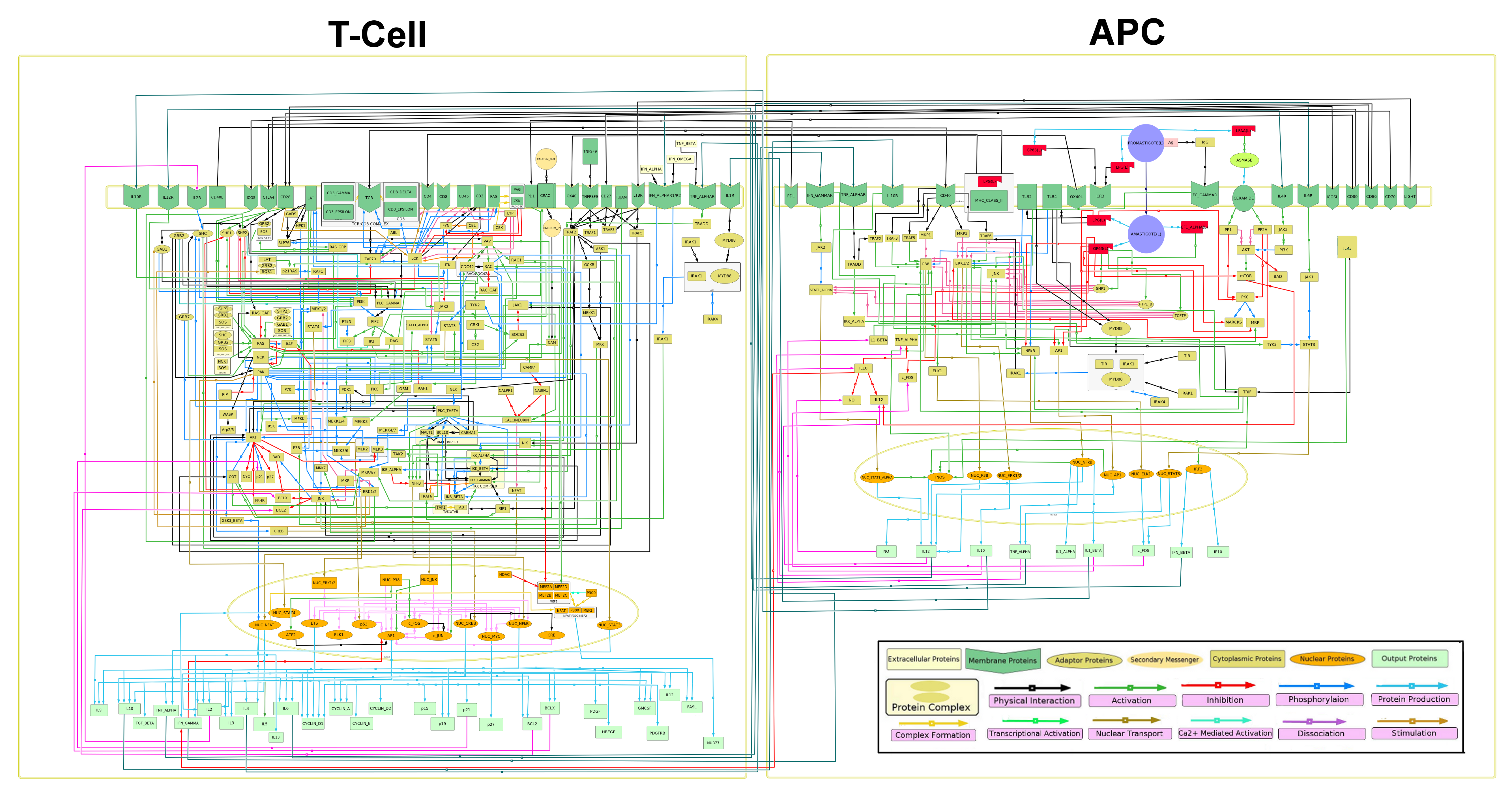

Supplement: Supplementary file 4 — Comprehensive diagram of T-cell, APC and Leishmania pathogenic protein-protein interaction network. The diagram presents an integrated view of the T-cell and APC interaction signaling pathway during Leishmania infection. The different molecules involved in the signaling cascade have been color coded according to its type and cellular location. The molecules colored as red signify the Leishmania antigen molecules. The interaction lines have been color coded according to the type of chemical reaction such as phosphorylation (blue), inhibition (red), activation (green) etc. (TIF 5212 kb) [file 13637_2015_32_MOESM4_ESM.tif]

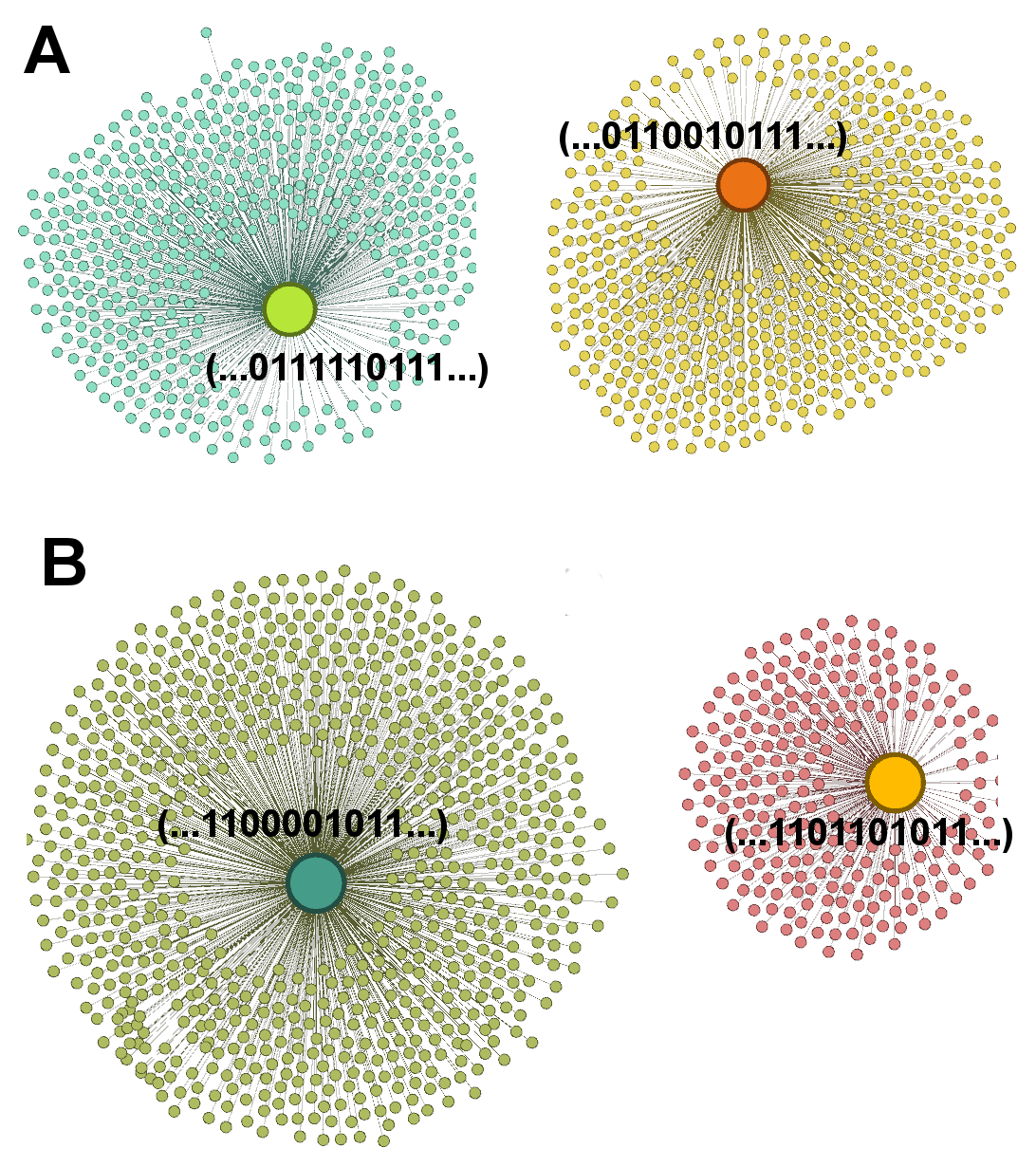

Supplement: Supplementary file 5 — Attractor analysis of the uninfected and infected scenarios under the differential activation of the splicing factors. (A) In the uninfected scenario the system reach two stable steady state attractors, in which the expressions of IFN_BETA, IL10, IL12, IL1_ALPHA, IL1_BETA, INOS, IP10, NO, TNF_ALPHA and C_FOS proteins are (0111110111) or (0110010111). (B) In the infected scenario, the system reach two stable steady state attractors namely (1100001011) and (1101101011), respectively. (TIF 339 kb) [file 13637_2015_32_MOESM5_ESM.tif]
